# What does collaborative healthcare for people with musculoskeletal-related conditions look like? A scoping review

**DOI:** 10.1186/s12891-025-08814-6

**Published:** 2025-07-04

**Authors:** James Roberts, Sinead Delaney, Adrian Mallows, Bradley Stephen Neal

**Affiliations:** 1 School of Sport, Rehabilitation and Exercise Sciences, University of Essex, Colchester, Essex C04 3SQ UK; 2 Pure Sports Medicine, London, UK; 3 Sports and Exercise Medicine, Queen Mary University of London, London, E1 4DG UK

**Keywords:** Scoping review, Self-management support, Shared decision-making, Education, Outcome measures

## Abstract

**Background:**

Managing musculoskeletal-related conditions usually benefits from involving patients in their care. Collaborative approaches support better health outcomes and enable individuals to take a more active role. However, the musculoskeletal literature remains unclear on how collaboration is reported and measured, as well as the factors that facilitate or inhibit it.

**Objectives:**

Summarise the components of the King’s Fund shared responsibility for health framework published in the musculoskeletal-related literature, and map the barriers, facilitators, and outcome measures of these components.

**Design:**

Scoping review.

**Method:**

Five electronic databases were searched. Two independent reviewers screened titles and abstracts followed by full texts to determine eligibility. Data relevant to the objective of the review were extracted independently by the lead author and ranked by frequency count.

**Results:**

The search returned 4,101 items, with 140 eligible for inclusion. The most frequently reported components of collaborative healthcare were self-management, education, and shared decision-making. Collaborative approaches including peer support and education, working with communities, designing services in partnership, personal budgets, patient leaders, and involving family/carers were modestly reported. Seventeen barriers and thirty-six facilitators to collaborative healthcare were identified and mapped to patient, clinician, and system-level organisational factors, with seventy-three outcome measures across eight constructs reported.

**Conclusions:**

The current musculoskeletal literature sparsely covers the range of collaborative approaches outlined by the King’s fund shared responsibility for health framework. More research is required to support the implementation and measurement of collaborative approaches to healthcare when managing musculoskeletal-related conditions.

**Clinical trial number:**

Not applicable.

**Supplementary Information:**

The online version contains supplementary material available at 10.1186/s12891-025-08814-6.

## Introduction

Musculoskeletal-related conditions are a major healthcare challenge for individuals and societies, with 17.1% of adults in England currently living with one [1]. Musculoskeletal-related conditions are the largest contributor to years lived with disability; negatively impacting quality of life and ability to work [2]. Musculoskeletal-related conditions also increase the risk of other long-term conditions [3], including obesity, cardiovascular disease, and depression. As it is predicted the prevalence of musculoskeletal-related conditions will continue to increase [4, 5], optimising approaches to support people experiencing them is urgently required.

One approach detailed in the National Health Service (NHS) long-term plan, published to ensure the sustainability and improvement of healthcare services in England, is to have a stronger focus on collective accountability for enhancing health and care between patients, the public, and the NHS [6]. This cultural shift proposed by The King’s Fund, a leading independent health think tank in England, is termed ‘shared responsibility for health’ [6, 7]. This does not involve delegating work but instead looks to emphasise the collective accountability of individuals and communities alongside formal systems. Recognising that some people may find this more challenging than others, tailored support is necessary to address varying needs. Approaches include promoting self-management, health coaching, information sharing, peer support, designing services in partnership, personal budgets, shared decision-making, greater involvement of families/carers, and working with communities [7]. These strategies require collaboration; promoting partnerships between people and healthcare professionals, empowering patients to actively participate in their care and decision-making. The benefits of supporting greater patient involvement in other long-term health conditions include reduced healthcare consumption, access to a greater variety of services, and improved outcomes, and is highly valued by patients [8–12].

Clinicians working in a musculoskeletal setting report a positive attitude towards working collaboratively with patients [13]. However, they often struggle to fully embrace patients taking greater control over decision-making [14, 15], meaning collaborative approaches are not always adopted [16, 17]. Implementation of collaborative healthcare requires organisational-level involvement to support delivery, monitoring, and evaluation [18]. If performed poorly, collaborative healthcare is associated with higher rates of excessive healthcare consumption and poorer patient-reported health outcomes [19].

For people with musculoskeletal-related conditions, the strategies reported in the literature to enable people to take greater control of their health are unknown [22, 23]. There is also a clear need to understand the barriers and facilitators clinicians face to adopting a collaborative approach to musculoskeletal healthcare. This scoping review aimed to summarise the components of the King’s Fund shared responsibility for health framework described and published in the musculoskeletal-related literature, and map the barriers, facilitators, and outcome measures of these components.

## Methods

### Protocol and registration

Our final protocol was registered prospectively with the Open Science Framework on 17/01/2023 (https://osf.io/872as/?view_only=d5cfd377d8c34b19b4c1d420387f0862) and developed using the preferred reporting items for systematic reviews and meta-analysis protocols extension for scoping reviews reporting guidelines (PRISMA – ScR; [20–22]).

### Eligibility criteria

The population, concept, context mnemonic (PCC) was used to guide a clear title and identify the main concepts for this scoping review [22], mapped to the King’s Fund shared responsibility for health framework. [7].

### Population

Any data source involving or specific to adults (aged ≥ 18) living with a musculoskeletal-related condition was eligible for inclusion.

### Concept

Eligible data sources were required to focus on specific dimensions of the King’s Fund's shared responsibility for health framework [7]. These methods (shared-decision making, self-management support, health coaching and personalised care planning, information sharing, peer support and education, designing services in partnership, patients as leaders, personal budgets, the role of carers and families and working with communities) have been reported to maximise patient expertise by facilitating more active and collaborative involvement in healthcare decisions.

### Context

All public settings of primary, intermediate, secondary, and private healthcare, all healthcare professionals involved in the management of people with musculoskeletal-related conditions, and all geographic locations, were eligible for inclusion.

### Types of evidence sources

Heterogenous data sources were eligible to reflect the broad nature of our research question [22]. All peer-reviewed methods were eligible to capture contributions from people living with musculoskeletal-related conditions, health care providers, and/or service managers, including qualitative, quantitative, and mixed methods. To capture any emerging evidence that may highlight current or proposed for implementation, non-peer-reviewed methods were also eligible (e.g., conference abstracts). Any sources of evidence that synthesised multiple data sources were excluded (e.g., book chapters, systematic reviews, Delphi studies or narrative syntheses).

### Information sources

We performed an initial scoping search in Medline and Web of Science. An analysis of text words within titles and abstracts was used to identify any additional search terms to be included in the final search (see Table 1), with assistance from an academic support librarian [22]. A comprehensive literature search with no date restrictions was subsequently performed in Medline, CINAHL, SPORTDiscus, American Psychiatric Association, and Web of Science on 17/2/2023. For pragmatic reasons, studies published in languages other than English were excluded.

**Table 1:** Search terms

	Search terms
S1	Collaborative healthcare OR patient involvement OR self management OR health coaching OR personalised care OR information sharing OR patient education OR peer support OR peer group OR service design OR personal budget OR shared decision making OR community integration OR community medicine OR therapeutic community OR social prescribing
S2	Physiotherap* or physical therap* or rehabilitat*
S3	Musculoskeletal
S4	S1 AND S2 AND S3

### Selection for sources of evidence

One reviewer (JR) exported all identified data sources into Covidence (Melbourne, Victoria, Australia), where duplicates were automatically removed. Two independent reviewers (JR, SD) initially screened 25 items as a pilot testing process and discussed the results to increase consistency. All remaining titles and abstracts, followed by full texts, were subsequently screened by the same two independent reviewers. Disagreements were initially resolved by consensus, and a third reviewer (BN) was available but not required.

### Data charting and items

Once all eligible data sources were screened against the eligibility criteria, we extracted key details into a pre-populated data charting table (Microsoft Excel for Windows, Microsoft Corporation, Washington, USA) with key headings relevant to the objective of the review (title and author, publication year, population, context when able or country of origin, methods, and the concept explored). Identified barriers and facilitators to collaborative healthcare and any outcome measures used were also extracted. Data charting was conducted by a single reviewer (JR) and second checked for accuracy by a second reviewer (SD).

### Analysis of evidence and presentation of results

Frequency counts of the reviewed concepts were depicted in tabular form to summarise the components of the King’s Fund shared responsibility for health framework reported in the musculoskeletal literature. Data are presented in tables and ranked by frequency count. An inductive process by a single author (JR) was used to identify and map strategies reported as either barriers or facilitators, with a new heading created and a frequency recorded for each novel characteristic. Barriers and facilitators were then mapped inductively relative to service, clinician, or patient when reported, with the five most frequent barriers of each component reported (when available or when frequencies equal to the top five ranked). Visualisations were produced using Wordcloud2 R (version 4.2.1, R Foundation for Statistical Computing, Vienna, Austria) to present results. An identical iterative process was followed to map all outcome measures reported for collaborative approaches, categorised by measured construct with total frequency count and reported by how they were deployed (e.g., patient- or observer-reported).

## Results

### Systematic search

**Fig. 1:**
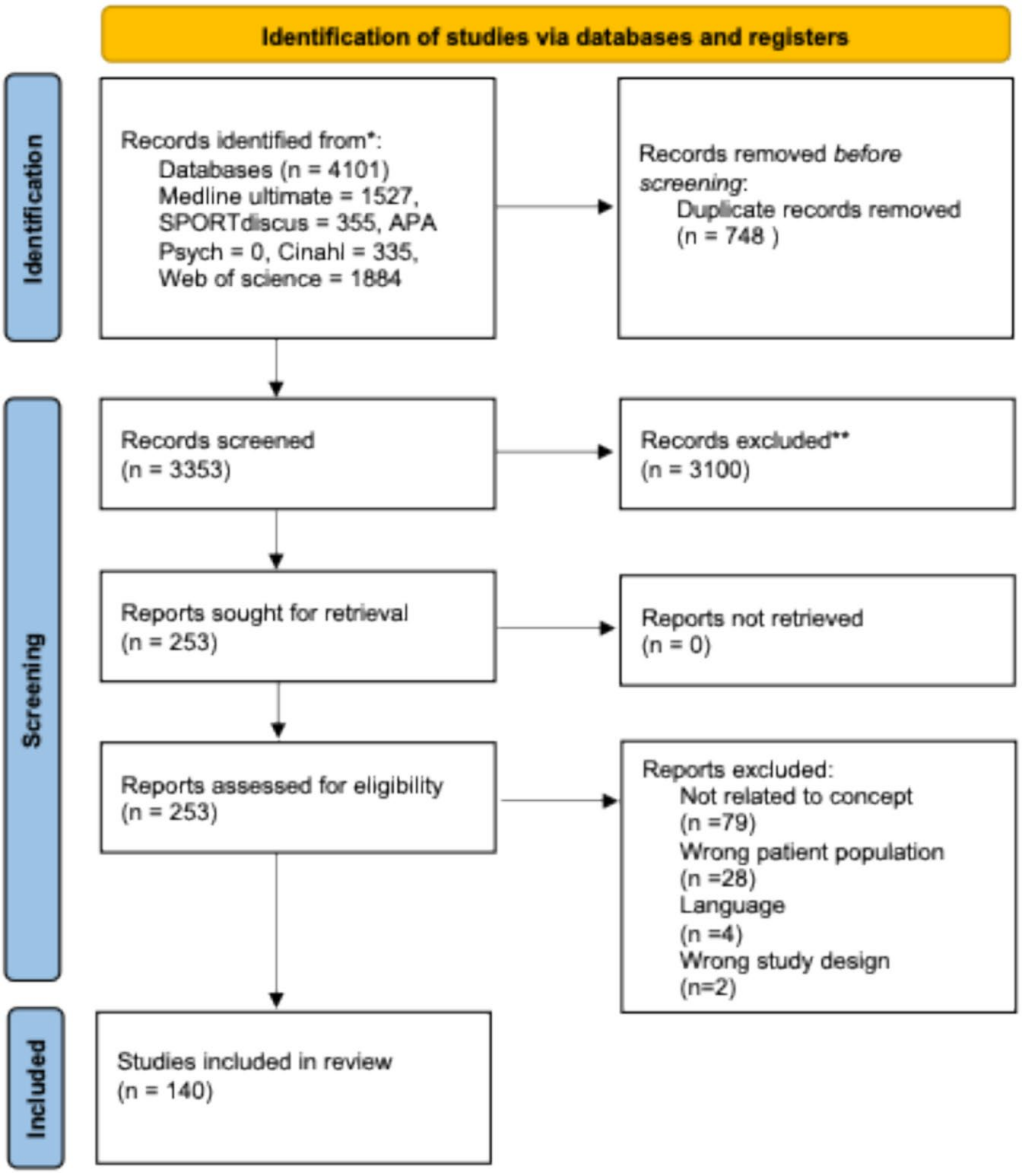
PRISMA flow diagram

Our systematic search returned 4,101 items; 3,353 after removing duplicates. 140 items were eligible for inclusion after screening titles and abstracts followed by full texts against our eligibility criteria (see Fig. 1). These 140 eligible items are included in the data charting table (supplementary file 1).

### Eligible items

Of the 140 eligible items, 44 were qualitative studies [12, 13, 23–64], 30 were randomised controlled trials (2 follow-ups and 2 secondary analyses) [65–94], 16 were observational studies (cohort/cross-sectional) [95–110], 12 were oral presentation abstracts [111–122],10 were feasibility studies [123–132], 9 were mixed-methods [133–141], 8 were pilot studies [142–149], 5 were case series or reports [150–154], 2 were content analyses [155, 156], 2 were quasi-experimental studies [157, 158], and 2 were intervention development study [159, 160].

### Components of the shared responsibility for health framework reported in the musculoskeletal literature

Within the shared responsibility for health framework, a breakdown of how the concepts reviewed were reported are detailed in Table 2.

**Table 2:** A frequency count for concepts reported

Concept	Frequency count
Self-management support	83 (59%)
Education	28 (20%)
Shared decision-making	12 (9%)
Peer support and education	6 (4%)
Health coaching and personalised care planning	4 (3%)
Working with communities	3 (2%)
Designing services in partnership	3 (2%)
The role of carers and families	1 (1%)
Patients as leaders	0 (0%)
Personal budgets	0 (0%)
Information sharing	0 (0%)

### Barriers to collaborative healthcare in musculoskeletal settings

**Fig. 2:**
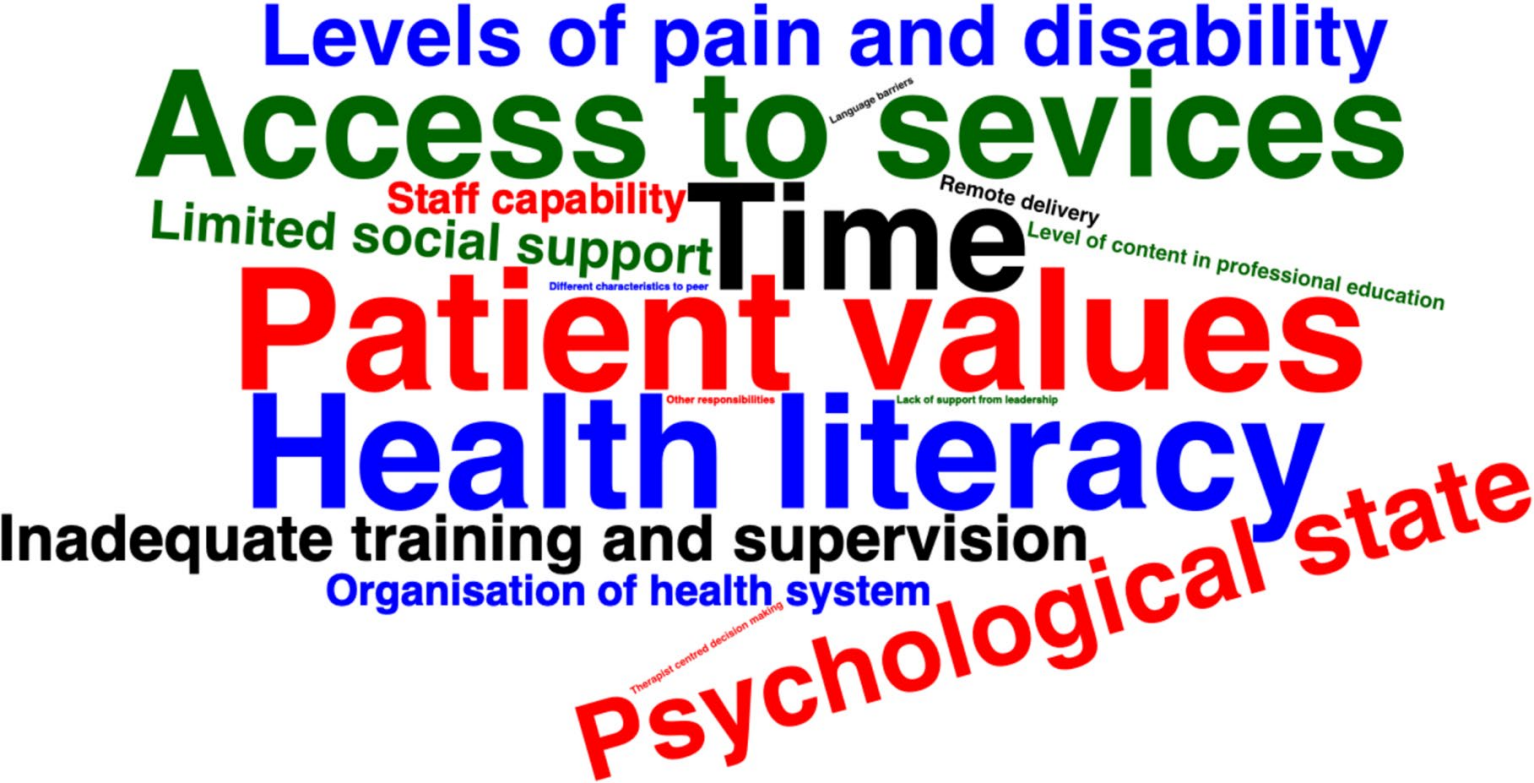
A word cloud representing the barriers to collaborative healthcare in musculoskeletal settings

The most frequent system-level barriers included limited time (*n* = 14; [26–39]), being unable to access services (transport, technology, internet; *n* = 14; [26, 29, 32, 34, 36, 38–46]), inadequate training and supervision from employers (*n* = 6; [29, 32, 41–43, 47]), working within a healthcare system that does not allow a biopsychosocial approach to healthcare (*n* = 4; [34, 39, 41, 47]), and inadequate training as part of undergraduate physiotherapy education (*n* = 2; [47, 48]). The most frequent individual clinician barriers included poor capability (*n* = 4; [34, 41, 42, 48]), and therapist-centred decision making (*n* = 1; [49]). The most frequent individual patient barriers included unique patient values (assuming a passive role, beliefs, expectations, uncertainty, religion; *n* = 16; [26, 32, 36, 38, 41, 42, 47, 48, 50–57]), low health literacy (*n* = 15; [28, 29, 37, 39, 40, 46, 48, 58–65]), negative psychological state (fear avoidance, self-efficacy, anxiety and depression, motivation, beliefs and expectations, coping and acceptance; *n* = 10; [26, 29, 34, 38, 48–50, 66–68]), high levels of pain and disability (*n* = 8; [26, 34, 38, 46, 50, 57, 69, 70]), and low social support (*n* = 5; [26, 32, 46, 50, 68]). These combined barriers are mapped visually in Fig. 2, where, the font size within the word cloud corresponds to the frequency of each barrier, with larger fonts indicating a higher frequency of occurrence and the full range of barriers identified are reported in supplementary file 2.

### Facilitators of collaborative healthcare in musculoskeletal settings

**Fig. 3:**
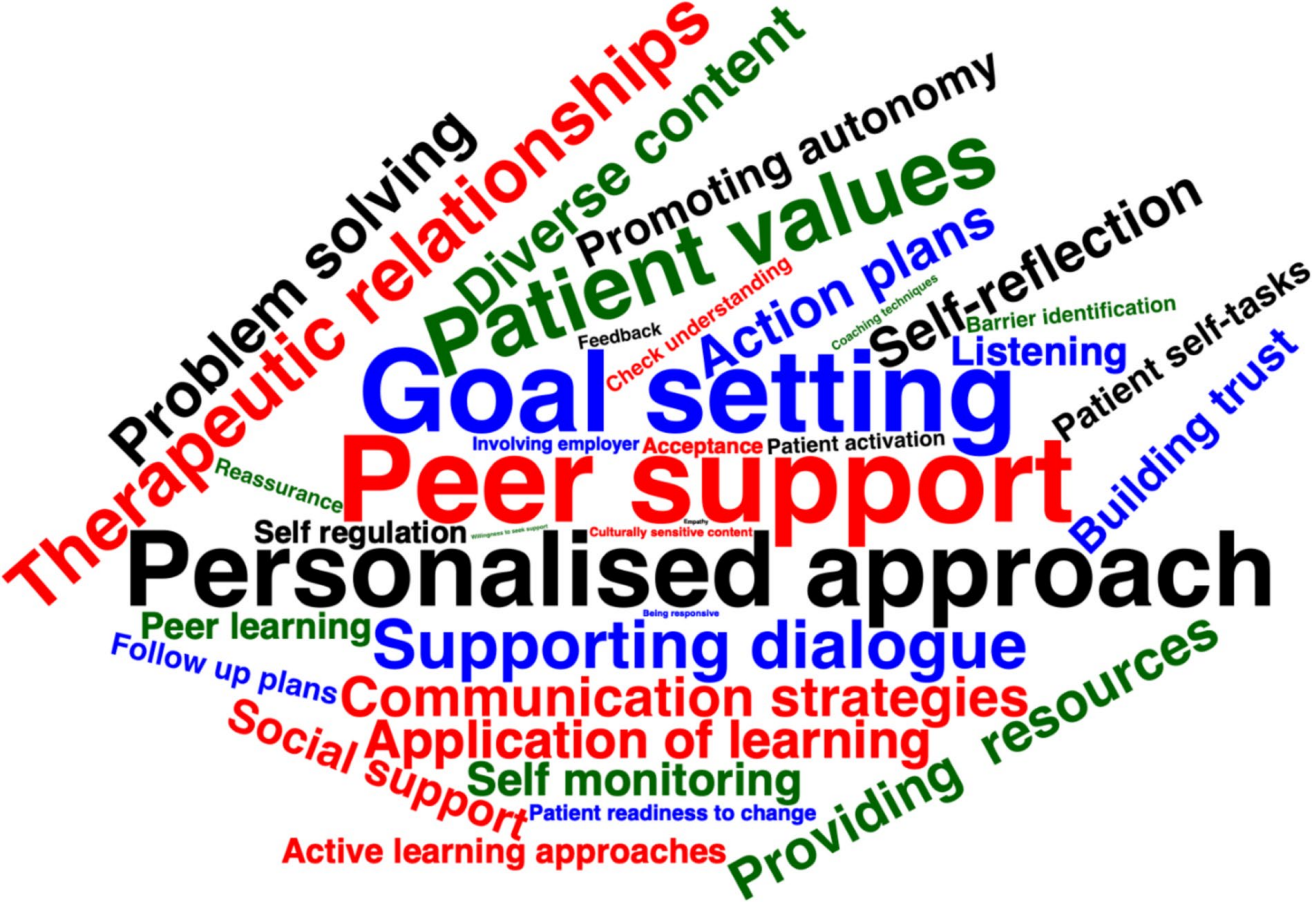
A word cloud representing the facilitators to collaboration within a musculoskeletal setting

No system-level facilitators were reported. The most frequent clinician facilitator was the role and use of goal setting (*n* = 37; [30, 35, 40, 42–44, 51, 60, 64, 66, 70–96]), personalising care (*n* = 33; [26, 35, 36, 39–41, 43, 50, 63, 64, 76, 77, 81, 86, 86–90, 92, 95, 97–109]), developing working alliance, (*n* = 24; [27, 35, 39, 40, 42, 43, 45, 47, 48, 51, 54, 56, 63, 70, 73, 75, 77, 83, 91, 92, 109–112]) supporting dialogue, (*n* = 22; [56, 57, 59, 61, 63, 71, 76, 77, 80, 83, 84, 86, 92, 101, 109, 112–118]) and problem solving (*n* = 20; [41, 44, 48, 51, 54, 75–77, 81–84, 88, 91, 96, 112, 119–122]). The patient's role as a facilitator was highlighted through peer support (*n* = 39; [28, 31, 70, 77–79, 83, 86, 91–93, 95, 38, 97, 102, 107, 108, 111, 114, 117, 120, 121, 123, 40, 124–131, 44, 52, 53, 59, 66, 68]), unique patient values (beliefs, treatment options, preferences and illness perceptions, priorities, concerns supported; *n* = 29; [31, 35, 73, 74, 78, 86, 92, 95, 96, 100, 104, 107, 41, 111–113, 116, 125, 130, 132–134, 43, 44, 46, 51, 54, 57, 63]), self-reflection (*n* = 18; [43, 45, 106, 116, 122, 123, 125, 127, 135, 136, 53, 56, 67, 84, 85, 91, 92, 96]) and peer learning (sharing experiences and strategies; *n* = 12; [28, 54, 123, 132, 70, 82, 86, 93, 107, 111, 117, 121]). The combined facilitators are mapped visually in Fig. 3, ranked by frequency, with the full range of identified facilitators are reported in supplementary file 3.

### Measures reported in musculoskeletal settings

**Fig. 4:**
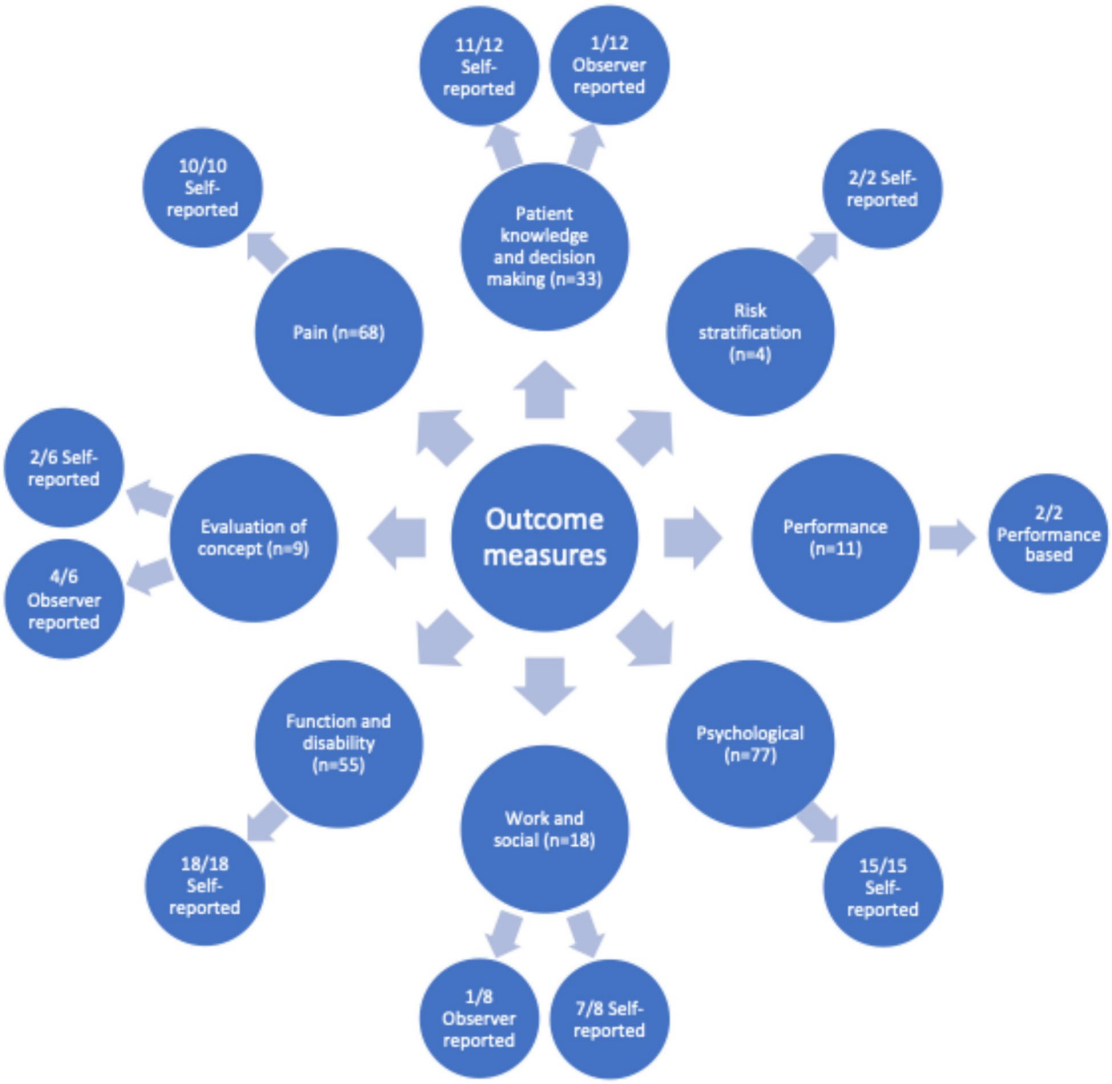
Spider diagram mapping outcome measures reported within included studies

Seventy-three outcome measures were identified in the included data sources, and iteratively grouped into eight categories: pain; function and disability; psychological factors; work and social factors, evaluation of concept; patient knowledge and decision making; performance; and risk stratification. The combined outcome measures, categorised by measured construct with total frequency count and method of deployment, are presented visually in Fig. 4. A further breakdown of the specific measures are reported in the tables presented in supplementary file 4.

## Discussion

The collaborative healthcare strategy most frequently reported in the musculoskeletal literature was supported self-management. This is recommended for the management of musculoskeletal-related conditions [162] and can aid people in developing the knowledge, skills, and confidence to actively manage their condition [163]. Delivery approaches to achieve supported self-management include group interventions facilitated by healthcare professionals, one-to-one sessions, and technological interventions (e.g., online self-help support). Group interventions led by healthcare professionals have demonstrated successful fostering of supported self-management in the short-, medium-, and long-term [164]. However, further understanding of effectiveness is needed to ensure that this approach supports the diverse needs of patients with musculoskeletal-related conditions, as it may increase demands on some individuals and result in poorer outcomes [165]. Other frequently reported components included education and shared decision-making, which are supported by NHS England to personalise care [166–168]. Whilst education is a common component of supported self-management programmes [169], it was described as a discrete component in the reviewed literature, with education aiming to increase understanding and empower people in managing their condition [170]. Recommendations for the delivery of these approaches have been outlined to support best practice [13, 169–173], but evidence of the effectiveness on long-term outcomes in the musculoskeletal literature is currently limited [174–176]. Despite the high frequency of these approaches reported in this review, healthcare professionals experience challenges when implementing them [14, 15, 177–181], highlighting that further support is required to make them a part of routine care.

Reported patient-related barriers to collaborative healthcare included unique values, low health literacy, negative psychological factors, and high levels of pain and disability, which aligns with previous studies [182–186]. As well as inhibiting collaboration, these identified barriers led to challenges in navigating healthcare systems and poorer musculoskeletal-related health outcomes [161, 182–185, 187–189]. Healthcare professionals have previously been identified as a barrier to collaboration and similar findings were identified in this review; with reduced capability, knowledge, and negative attitudes to support greater patient involvement highlighted [178, 190–192]. To make a reality of the cultural change proposed by the King’s Fund shared responsibility for health framework, current and future healthcare professionals need to be able to identify and support patient's capabilities and motivation to be involved in decisions and develop approaches to equip people to take greater responsibility. A further barrier to collaboration identified both in this review and the previous literature was access to services and limited consultation time [54], however innovative community-based projects to transform the delivery of care and subsequent barriers are reported in other health conditions, suggesting new ways of working [193]. To achieve the maximum benefits healthcare workers require system-level support to design and deliver services to support structural, cultural, and attitudinal changes or risk falling short of reaching the ambition set out by the King’s fund and the NHS long-term plan [6, 7, 168].

A previous review identified physiotherapists as working collaboratively by involving patients in decision-making, promoting dialogue, and being responsive to individual needs [194], which aligns with key facilitators found in this review. Approaches reported frequently to facilitate patient involvement included goal setting, problem-solving, action planning, and coping planning, and have been encouraged in practice to support interpersonal collaboration and the development of the knowledge, skills, and confidence to self-manage [163, 166, 167, 195, 196]. Personalised approaches were highlighted in this review to facilitate collaboration; providing opportunities to understand “what matters to you”, rather than “what is the matter with you” [197], reflecting the focus on personalisation in the NHS long-term plan. Peer support was reported as a frequent facilitator and strategies to encourage greater dialogue with peers can support social connection, feelings of isolation, coping skills, and attitudinal change in people with musculoskeletal-related conditions [190, 198]. Despite a wide range of facilitators of collaboration reported frequently in this review their implementation in practice remains unclear. Evidence of effectiveness is required to ensure these facilitators of greater involvement in people with musculoskeletal pain are beneficial.Within the shared responsibility for health framework

The constructs most frequently measured related to function and disability, psychological factors and pain; with patient-reported outcome measures most frequently deployed. Measures of collaborative approaches to support self-management and identify patient involvement have been developed and encouraged [199, 200], but this review identified a low-frequency count for any measure to identify patients’ knowledge and decision-making and subsequent capabilities or motivation to be involved, alongside low reporting of measures to observe the collaborative approach. Similar findings of uncertainty relevant to measurement to support implementation have been reported in attempts to capture personalised care [201], highlighting the gap in the promotion of these approaches to what people may experience [202]. If the management of musculoskeletal-related conditions benefits from involving patients in their care greater certainty is required on how to measure its implementation and effectiveness to ensure the sustainability of greater patient involvement.

### Limitations

The breadth of our question may have hindered our ability to map the whole body of evidence. To identify a wider range of approaches; evidence from the grey literature, professional bodies, or websites may have identified innovative data relevant to our objectives. We decided not search for these resources due to the anticipated large volume of data extraction identified following the initial scoping search, the broad nature of the question, and multiple objectives [203]. We instead conducted a comprehensive literature search across multiple databases to capture contributions from a wide range of peer-reviewed methods.

Another limitation was the potential bias toward studies involving the physiotherapy profession. Although no studies were excluded based on profession, the search was developed to reflect the increasing role that physiotherapists are having across the triage and management pathways of musculoskeletal-related conditions [204, 205]. We acknowledge that this may have limited the inclusion of literature involving other healthcare professionals and subsequent generalisability across all musculoskeletal-related care pathways. As only 95 of the 140 (68%) included studies involved physiotherapy in the population or concept, we are confident that other professions have been captured within our chosen search.

### Implications for future work

Future studies in people with musculoskeletal-related conditions should look to capture components of the King’s Fund shared responsibility for health framework less frequently researched [206]. This should include peer support and education, health coaching and personalised care planning, and working with communities. These components have been developed across other long-term conditions, comparing favourably to medical settings in psychological and physical constructs [193, 207–216], but robust evidence of implementation and outcomes is limited in musculoskeletal healthcare. Key barriers and facilitators to greater collaboration between the healthcare system and people with musculoskeletal pain were identified and largely aligned with previous literature. It is unknown how well these barriers are considered or how well facilitators are delivered and if they can overcome the barriers to collaboration. To understand how well these approaches are delivered, an ability to measure the concept is required, and this review highlighted that the evaluation of collaborative approaches is under-reported, and measures to appraise the capabilities and confidence of people with musculoskeletal-reported conditions are limited. Future research should look to understand the effectiveness and implementation of collaborative approaches to make a reality of the cultural shift proposed by the King’s Fund [7].

## Conclusion

Components of the King’s Fund shared responsibility for health framework frequently reported in the published musculoskeletal-related literature include self-management support, education, and shared decision-making, but many are reported infrequently. To facilitate collaborative healthcare, barriers including access to services, time constraints, low health literacy, high pain and disability, psychological factors, individual values, staff capability, and therapist-led decision-making should be addressed. Goal setting, personalising care, developing working alliances, promoting dialogue, and problem-solving are reported frequently and should facilitate the implementation of collaborative healthcare. This review outlines opportunities for strategies to move away from the paternalistic medical model of ‘clinician knows best’ and ensure that the visions of the NHS long-term plan can become ‘business as usual’.

## Supplementary Information

Supplementary Material 1

Supplementary Material 2

Supplementary Material 3

Supplementary Material 4

## Data Availability

All available data is included in the published manuscript or related supplementary files.

## Abbreviations

NHS: National Health Service
PRISMA – ScR: Preferred reporting items for systematic reviews and meta-analysis protocols extension for scoping reviews
PCC: Population, concept, context mnemonic

## References

[1] Musculoskeletal health: trends, risk factors and disparities in England [Internet]. [cited 2022 Dec 2]. URL:https://fingertips.phe.org.uk/static-reports/musculoskeletal-conditions/musculoskeletal-health-trends-risk-factors-and-disparities-in-england.html?s=09

[2] Versus Arthritis. the State of Musculoskeletal Health 2024. 2024;3–4. https://versusarthritis.org/media/embkyapu/the-state-of-musculoskeletal-health-2024.pdf.

[3] Williams A, Kamper SJ, Wiggers JH, O’Brien KM, Lee H, Wolfenden L, et al. Musculoskeletal conditions may increase the risk of chronic disease: A systematic review and meta-analysis of cohort studies. BMC Med [Internet]. 2018;16(1):1–9. 10.1186/s12916-018-1151-2. 10.1186/s12916-018-1151-2 PMC6154805 30249247

[4] Hartvigsen J, Hancock MJ, Kongsted A, Louw Q, Ferreira ML, Genevay S, et al. What low back pain is and why we need to pay attention. Lancet. 2018 Jun 9;391(10137):2356–67. 10.1016/S0140-6736(18)30480-X. Cited 2024 Mar 21 10.1016/S0140-6736(18)30480-X 29573870

[5] Gill TK. Global, regional, and national burden of other musculoskeletal disorders, 1990–2020, and projections to 2050: a systematic analysis of the Global Burden of Disease Study 2021. Lancet Rheumatol. 2023;5:e670–82. 10.1016/S2665-9913(23)00232-1. Cited 2024 Mar 21 10.1016/S2665-9913(23)00232-1 PMC10620749 37927903

[6] The NHS Long Term Plan. 2019. Cited 2023 Nov 20. https://www.longtermplan.nhs.uk/wpcontent/uploads/2019/08/nhs-long-term-plan-version-1.2.pdf.

[7] Ham C, Charles A, Wellings D. The Kings Fund. 2018. Shared responsibility for health | The King’s Fund. URL:https://www.kingsfund.org.uk/publications/shared-responsibility-health. Cited 2022 Dec 13

[8] Batalden P. Getting more health from healthcare: quality improvement must acknowledge patient coproduction-an essay by Paul Batalden. 2018; 10.1136/bmj.k3617. Cited 2022 Nov 25

[9] Kennedy A, Rogers A, Bower P. Support for self care for patients with chronic disease. BMJ Br Med J. 2007 Nov 11;335(7627):968. 10.1136/BMJ.39372.540903.94. Cited 2022 Dec 6 10.1136/bmj.39372.540903.94 PMC2071971 17991978

[10] Hibbard J, Gilburt H. Supporting people to manage their health An introduction to patient activation. 2014. https://assets.kingsfund.org.uk/f/256914/x/d5fbab2178/supporting_people_manage_their_health_2014.pdf.

[11] Deeny S, Thorlby R, Steventon A. Briefing: Reducing emergency admissions: unlocking the potential of people to better manage their long-term conditions. 2018. https://www.health.org.uk/sites/default/files/Reducing-Emergency-Admissions-long-term-conditions-briefing.pdf.

[12] Bernhardsson S, Samsson KS, Johansson K, Öberg B, Larsson MEH. A preference for dialogue: exploring the influence of patient preferences on clinical decision making and treatment in primary care physiotherapy. Eur J Physiother. 2019 Apr 3;21(2):107–14. 10.1080/21679169.2018.1496474. Cited 2023 Feb 6

[13] Hoffmann T, Gibson E, Barnett C, Maher C. Shared decision making in Australian physiotherapy practice: A survey of knowledge, attitudes, and self-reported use. PLoS One. 2021;16(5):e0251347. 10.1371/journal.pone.0251347. 10.1371/journal.pone.0251347 PMC8136718 34014934

[14] Grenfell J, Soundy A. People’s Experience of Shared Decision Making in Musculoskeletal Physiotherapy: A Systematic Review and Thematic Synthesis. Behav Sci (Basel). 2022 Jan 1;12(1). 10.3390/BS12010012/S1. Cited 2022 Dec 6 10.3390/bs12010012 PMC8773142 35049623

[15] Mathijssen EGE, Van Den Bemt BJF, Wielsma S, Van Den Hoogen FHJ, Vriezekolk JE. Exploring healthcare professionals’ knowledge, attitudes and experiences of shared decision making in rheumatology. RMD Open. 2020 Jan 1;6(1):e001121. 10.1136/RMDOPEN-2019-001121. Cited 2023 Jan 13 10.1136/rmdopen-2019-001121 PMC7046943 31958279

[16] Holden MA, Nicholls EE, Young J, Hay EM, Foster NE. UK-based physical therapists’ attitudes and beliefs regarding exercise and knee osteoarthritis: findings from a mixed-methods study. Arthritis Rheum. 2009 Nov 15;61(11):1511–21. 10.1002/ART.24829. Cited 2022 Dec 6 10.1002/art.24829 19877105

[17] Jones LE, Roberts LC, Little PS, Mullee MA, Cleland JA, Cooper C. Shared decision-making in back pain consultations: an illusion or reality? Eur Spine J. 2014;23 Suppl 1(Suppl 1). 10.1007/S00586-014-3187-0. Cited 2022 Dec 6 10.1007/s00586-014-3187-0 PMC3946094 24477377

[18] NICE. Recommendations | Transition between inpatient hospital settings and community or care home settings for adults with social care needs | Guidance | NICE. NICE Guidel. 2016;(December 2016):1–33. https://www.nice.org.uk/guidance/ng27.

[19] Hughes TM, Merath K, Chen Q, Sun S, Palmer E, Idrees JJ, et al. Association of shared decision-making on patient-reported health outcomes and healthcare utilization. Am J Surg. 2018 Jul 1;216(1):7–12. 10.1016/J.AMJSURG.2018.01.011. Cited 2022 Dec 6 10.1016/j.amjsurg.2018.01.011 29395026

[20] Peters MDJ, Godfrey CM, Khalil H, McInerney P, Parker D, Soares CB. Guidance for conducting systematic scoping reviews. Int J Evid Based Healthc. 2015 Sep 1;13(3):141–6. 10.1097/XEB.0000000000000050. Cited 2022 Apr 29 10.1097/XEB.0000000000000050 26134548

[21] Tricco AC, Lillie E, Zarin W, O’Brien KK, Colquhoun H, Levac D, et al. PRISMA Extension for Scoping Reviews (PRISMA-ScR): Checklist and Explanation. Ann Intern Med. 2018 Oct 2;169(7):467–73. 10.7326/M18-0850. Cited 2022 Apr 29 10.7326/M18-0850 30178033

[22] JBI Manual for Evidence Synthesis - JBI Global Wiki. URL:https://jbi-global-wiki.refined.site/space/MANUAL/4687737/11.2.2+Developing+the+title+and+question. Cited 2022 Dec 13

[23] Cridland K, Pritchard S, Rathi S, Malliaras P. ‘He explains it in a way that I have confidence he knows what he is doing’: A qualitative study of patients’ experiences and perspectives of rotator-cuff-related shoulder pain education. Musculoskeletal Care. 2021Jun 1;19(2):217–31. 10.1002/msc.1528. 33258225 10.1002/msc.1528

[24] McCluskey S, De Vries H, Reneman M, Brooks J, Brouwer S. “I think positivity breeds positivity”: A qualitative exploration of the role of family members in supporting those with chronic musculoskeletal pain to stay at work Knowledge, attitudes, behaviors, education, and communication. BMC Fam Pract. 2015 Jul 22;16(1). 10.1186/s12875-015-0302-1 10.1186/s12875-015-0302-1 PMC4509776 26198218

[25] Acker R, Swain N, Perry M, Wassinger C, Sole G. ‘Thinking about pain in a different way’: Patient perspectives of a neuroscience-informed physiotherapy programme for rotator cuff-related shoulder pain. Musculoskelet Sci Pract. 2023Feb;1:63. 10.1016/j.msksp.2022.102691. 10.1016/j.msksp.2022.102691 36538858

[26] Andersen LN, Kohberg M, Herborg LG, Søgaard K, Roessler KK. “Here we’re all in the same boat” - A qualitative study of group based rehabilitation for sick-listed citizens with chronic pain. Scand J Psychol. 2014;55(4):333–42. 10.1111/sjop.12121. 24730653 10.1111/sjop.12121

[27] Franklin ZC, Smith NC, Fowler NE. A qualitative investigation of factors that matter to individuals in the pain management process. Disabil Rehabil. 2016Sep 10;38(19):1934–42. 10.3109/09638288.2015.1107782. 26728636 10.3109/09638288.2015.1107782

[28] Semedo B, Stålnacke BM, Stenberg G. A qualitative study among women immigrants from Somalia–experiences from primary health care multimodal pain rehabilitation in Sweden. Eur J Physiother. 2020Jul 3;22(4):197–205. 10.1080/21679169.2019.1571101.

[29] Bair MJ, Matthias MS, Nyland KA, Huffman MA, Stubbs DWL, Kroenke K, et al. Barriers and facilitators to chronic pain self-management: A qualitative study of primary care patients with comorbid musculoskeletal pain and depression. Pain Med. 2009Oct;10(7):1280–90. 10.1111/j.1526-4637.2009.00707.x. 19818038 10.1111/j.1526-4637.2009.00707.x PMC2884223

[30] Skúladóttir H, Gunnarsdóttir TJ, Halldórsdóttir S, Sveinsdóttir H, Holden JE, Björnsdóttir A. Breaking the vicious circle: Experiences of people in chronic pain on the pain rehabilitation journey. Nurs Open. 2020Sep 1;7(5):1412–23. 10.1002/nop2.512. 32802361 10.1002/nop2.512 PMC7424485

[31] Dragesund T, Øien AM. Developing self-care in an interdependent therapeutic relationship: patients’ experiences from Norwegian psychomotor physiotherapy. Physiother Theory Pract. 2021;38(11):1656–66. 10.1080/09593985.2021.1875524. 33461377 10.1080/09593985.2021.1875524

[32] Moe RH, Haavardsholm EA, Grotle M, Steen E, Kjeken I, Hagen K, et al. Development of a brief multidisciplinary education programme for patients with osteoarthritis. Vol. 12, BMC Musculoskeletal Disorders. 2011. 10.1186/1471-2474-12-257 10.1186/1471-2474-12-257 PMC3262862 22077985

[33] Kelly M, Fullen BM, Martin D, Bradley C, McVeigh JG. eHealth interventions to support self-management: Perceptions and experiences of people with musculoskeletal disorders and physiotherapists - ‘eHealth: It’s TIME’: A qualitative study. Physiother Theory Pract. 2022;40(5):1011–21. 10.1080/09593985.2022.2151334. 36426843 10.1080/09593985.2022.2151334

[34] Brunnekreef JJ, Feleus A, Miedema HS, Staal JB, Hutting N. Experiences and needs of physiotherapists and exercise therapists regarding the management of working people with complaints of the arm, neck and shoulder (CANS): A focus group study. Musculoskelet Sci Pract. 2022Dec;1:62. 10.1016/j.msksp.2022.102644. 10.1016/j.msksp.2022.102644 35985147

[35] van den Heuvel C, van der Horst J, Winkelhorst E, Roelofsen E, Hutting N. Experiences, barriers and needs of physiotherapists with regard to providing self-management support to people with low back pain: A qualitative study. Musculoskelet Sci Pract. 2021Dec;1:56. 10.1016/j.msksp.2021.102462. 10.1016/j.msksp.2021.102462 34571401

[36] Barrett E, Hayes A, Kelleher M, Conroy C, Robinson K, O’Sullivan K, et al. Exploring patient experiences of participating in a group exercise class for the management of nonspecific shoulder pain. Physiother Theory Pract. 2018Jun 3;34(6):464–71. 10.1080/09593985.2017.1422208. 29297720 10.1080/09593985.2017.1422208

[37] Johansen SK, Maclachlan L, Hillier R, Taylor G, Mellor R, Rathleff MS, et al. Exploring patients’ and physiotherapists’ visions on modelling treatments and optimising self-management strategies for patellofemoral pain: A future workshop approach. In: Musculoskeletal Science and Practice. Elsevier Ltd; 2022. 10.1016/j.msksp.2022.102567 10.1016/j.msksp.2022.102567 35468529

[38] Lavender EC, Dusabe-Richards E, Anderson AM, Antcliff D, McGowan L, Conaghan PG, et al. Exploring the feasibility, acceptability and value of volunteer peer mentors in supporting self-management of osteoarthritis: a qualitative evaluation. Disabil Rehabil. 2022;44(21):6314–24. 10.1080/09638288.2021.1964625. 34498993 10.1080/09638288.2021.1964625 PMC9590401

[39] Shue SA, Mcguire AB, Matthias MS. Facilitators and Barriers to Implementation of a Peer Support Intervention for Patients with Chronic Pain: A Qualitative Study. Pain Med (United States). 2019Jul 1;20(7):1311–20. 10.1093/pm/pny229. 10.1093/pm/pny229 30481295

[40] Najem C, Wijma AJ, Meeus M, Cagnie B, Ayoubi F, Van Oosterwijck J, et al. Facilitators and barriers to the implementation of pain neuroscience education in the current Lebanese physical therapist health care approach: a qualitative study. Disabil Rehabil. 2023Jan;18:1–9. 10.1080/09638288.2023.2168076. 10.1080/09638288.2023.2168076 36655277

[41] Gustafsson M, Ekholm J, Öhman A. From shame to respect: Musculoskeletal pain patients’ experience of a rehabilitation programme, a qualitative study. J Rehabil Med. 2004May;36(3):97–103. 10.1080/16501970310018314. 15209451 10.1080/16501970310018314

[42] Chala MB, Miller J, Ghahari S, Wondie Y, Abebe A, Donnelly C. Health care providers’ understanding of self-management support for people with chronic low back pain in Ethiopia: an interpretive description. BMC Health Serv Res. 2022 Dec 1;22(1). 10.1186/s12913-022-07610-5 10.1186/s12913-022-07610-5 PMC8842538 35164738

[43] Meade LB, Bearne LM, Godfrey EL. “It’s important to buy in to the new lifestyle”: barriers and facilitators of exercise adherence in a population with persistent musculoskeletal pain. Disabil Rehabil. 2021;43(4):468–78. 10.1080/09638288.2019.1629700. 31242395 10.1080/09638288.2019.1629700

[44] Patel G, Walsh N, Gooberman-Hill R. Managing Osteoarthritis in Primary Care: Exploring Healthcare Professionals’ Views on a Multiple-Joint Intervention Designed to Facilitate Self-Management. Musculoskeletal Care. 2014Dec 1;12(4):199–209. 10.1002/msc.1074. 24840914 10.1002/msc.1074

[45] Rizzo J, Bell A. Mental models of adherence: parallels in perceptions, values, and expectations in adherence to prescribed home exercise programs and other personal regimens. Disabil Rehabil. 2019Sep 25;41(20):2412–20. 10.1080/09638288.2018.1466923. 29739240 10.1080/09638288.2018.1466923

[46] Wellman J, Murray L, Hebron C, Vuoskoski P. Pain Education in the Context of Non-Specific Low Back Pain: The Lived Experience of the Physiotherapist. An Interpretive Phenomenological Analysis. Musculoskeletal Care. 2020 Sep 1;18(3):271–300. 10.1002/MSC.1460. Cited 2024 May 2 10.1002/msc.1460 32293097

[47] King R, Robinson V, Elliott-Button HL, Watson JA, Ryan CG, Martin DJ. Pain reconceptualisation after pain neurophysiology education in adults with chronic low back pain: A qualitative study. Pain Res Manag. 2018;2018. 10.1155/2018/3745651 10.1155/2018/3745651 PMC6157134 30275918

[48] Bunzli S, Mcevoy S, Dankaerts W, O’sullivan P, O’sullivan K. Patient Perspectives on Participation in Cognitive Functional Therapy for Chronic Low Back Pain. 2016. 10.2522/ptj.20140570 10.2522/ptj.20140570 27013577

[49] Morris AL. Patients’ perspectives on self-management following a back rehabilitation programme. Musculoskeletal Care. 2004Aug;2(3):165–79. 10.1002/msc.68. 17041980 10.1002/msc.68

[50] Joelsson M, Bernhardsson S, Larsson MEH. Patients with chronic pain may need extra support when prescribed physical activity in primary care: a qualitative study. Scand J Prim Health Care. 2017Jan 2;35(1):64–74. 10.1080/02813432.2017.1288815. 28277047 10.1080/02813432.2017.1288815 PMC5361421

[51] Eiken AG, Nordanger D, Nes LS, Varsi C. Patients’ Experiences of Using an eHealth Pain Management Intervention Combined With Psychomotor Physiotherapy: Qualitative Study. JMIR Form Res. 2022 Mar 1;6(3). 10.2196/34458 10.2196/34458 PMC8968559 35293866

[52] Horler C, Hebron C, Martyn K. Personalizing education: The clinical reasoning processes of physiotherapists using education for the treatment of people with chronic low back pain. Physiother Theory Pract. 2022;38(3):412–21. 10.1080/09593985.2020.1765437. 32431203 10.1080/09593985.2020.1765437

[53] Hartholt E, Vuoskoski P, Hebron C. Physiotherapists’ lived experiences of decision making in therapeutic encounters with persons suffering from whiplash-associated disorder: A hermeneutic phenomenological study. Musculoskeletal Care. 2020Dec 1;18(4):519–26. 10.1002/msc.1496. 32677355 10.1002/msc.1496

[54] Hurley M, Sheldon H, Connolly M, Carter A, Hallett R. Providing easier access to community-based healthcare for people with joint pain: Experiences of delivering ESCAPE-pain in community venues by exercise professionals. Musculoskeletal Care. 2022Jun 1;20(2):408–15. 10.1002/msc.1584. 34375034 10.1002/msc.1584 PMC9487982

[55] Penney LS, Haro E. Qualitative evaluation of an interdisciplinary chronic pain intervention: Outcomes and barriers and facilitators to ongoing pain management. J Pain Res. 2019;12:865–78. 10.2147/JPR.S185652. 30881097 10.2147/JPR.S185652 PMC6402709

[56] Achten JPJ, Mooren-van der Meer S, Pisters MF, Veenhof C, Koppenaal T, Kloek CJJ. Self-management behaviour after a physiotherapist guided blended self-management intervention in patients with chronic low back pain: A qualitative study. Musculoskelet Sci Pract. 2022 Dec 1;62. 10.1016/j.msksp.2022.102675 10.1016/j.msksp.2022.102675 36332333

[57] Adams J, Lowe W, Protheroe J, Lueddeke J, Armstrong R, Russell C, et al. Self-management of a musculoskeletal condition for people from harder to reach groups: a qualitative patient interview study. Disabil Rehabil. 2019Dec 4;41(25):3034–42. 10.1080/09638288.2018.1485182. 30369265 10.1080/09638288.2018.1485182 PMC6913654

[58] Hutting N, Oswald W, Staal JB, Heerkens YF. Self-management support for people with non-specific low back pain: A qualitative survey among physiotherapists and exercise therapists. Musculoskelet Sci Pract. 2020Dec;1:50. 10.1016/j.msksp.2020.102269. 10.1016/j.msksp.2020.102269 33039797

[59] Osborn-Jenkins L, Roberts L. The advice given by physiotherapists to people with back pain in primary care. Musculoskelet Sci Pract. 2021Oct;1:55. 10.1016/j.msksp.2021.102403. 10.1016/j.msksp.2021.102403 34130069

[60] McIlroy S, Vaughan B, Crowe H, Bearne L. The experiences and acceptability of a novel multimodal programme for the management of fibromyalgia: A qualitative service evaluation. Musculoskeletal Care. 2022Sep 1;20(3):686–96. 10.1002/msc.1672. 35837789 10.1002/msc.1672 PMC9545101

[61] Pate JW, Tran E, Radhakrishnan S, Leaver AM. The importance of perceived relevance: a qualitative evaluation of patient’s perceptions of value and impact following a low-intensity group-based pain management program. Med. 2021Jan 1;57(1):1–12. 10.3390/medicina57010046. 10.3390/medicina57010046 PMC7826549 33430427

[62] Stern BZ, Njelesani J, Howe TH. Transitioning from hurting to healing: self-management after distal radius fracture. Disabil Rehabil. 2022;44(21):6277–86. 10.1080/09638288.2021.1962990. 34388959 10.1080/09638288.2021.1962990

[63] Lavender EC, Anderson AM, Dusabe-Richards E, Antcliff D, Kingsbury SR, Conaghan PG, et al. Understanding peer mentorship in supporting self-management of hip and knee osteoarthritis: A qualitative study of mentees’ perspectives. Musculoskeletal Care. 2022Mar 1;20(1):180–91. 10.1002/msc.1580. 34314551 10.1002/msc.1580 PMC9290819

[64] Parsons S, Harding G, Breen A, Foster N, Pincus T, Vogel S, et al. Will shared decision making between patients with chronic musculoskeletal pain and physiotherapists, osteopaths and chiropractors improve patient care? Fam Pract. 2012Apr 1;29(2):203–12. 10.1093/fampra/cmr083. 21982810 10.1093/fampra/cmr083

[65] Gustavsson C, von Koch L. A 9-year follow-up of a self-management group intervention for persistent neck pain in primary health care: A randomized controlled trial. J Pain Res. 2016Dec;30(10):53–64. 10.2147/JPR.S125074. 10.2147/JPR.S125074 PMC5221717 28115865

[66] Haugli, Liv M.D.*; Steen, Eldri R.N., M.Ed.*; Lærum, Even M.D., Ph.D.*; Finset, Arnstein Ph.D.†; Nygaard RPD. Agency Orientation and Chronic Musculoskeletal Pain: Effects of a Group Learning Program Based on the Personal Construct Theory. 10.1097/00002508-200012000-00002 10.1097/00002508-200012000-00002 11153782

[67] Cheng S, Chen-P, Chow Y, Law A, Lee J, Leung E, et al. An Exercise Cum Cognitive-Behavioral Intervention for Older Adults With Chronic Pain: A Cluster-Randomized Controlled Trial. J Consult Clin Psychol. 2022; 10.1037/ccp0000698.supp 10.1037/ccp0000698 35099206

[68] Malfliet A, Kregel J, Meeus M, Roussel N, Danneels L, Cagnie B, et al. Blended-Learning Pain Neuroscience Education for People With Chronic Spinal Pain: Randomized Controlled Multicenter Trial. Vol. 98, Physical Therapy. 2018. 10.1093/ptj/pzx092 10.1093/ptj/pzx092 29669079

[69] Werner EL, Storheim K, Lochting I, Wisloff T, Grotle M. Cognitive patient education for low back pain in primary care: A cluster randomized controlled trial and cost-effectiveness analysis. Spine (Phila Pa 1976). 2016 Mar 4;41(6):455–62. 10.1097/BRS.0000000000001268 10.1097/BRS.0000000000001268 26966970

[70] Hutting N, Bart Staal J, Engels JA, Heerkens YF, Detaille SI, Nijhuis-Van Der Sanden MWG. Effect evaluation of a self-management programme for employees with complaints of the arm, neck or shoulder: A randomised controlled trial. Occup Environ Med. 2015 Dec 1;72(12):852–61. 10.1136/oemed-2015-103089 10.1136/oemed-2015-103089 26359220

[71] Choudhry NK, Fifer S, Fontanet CP, Archer KR, Sears E, Bhatkhande G, et al. Effect of a Biopsychosocial Intervention or Postural Therapy on Disability and Health Care Spending among Patients with Acute and Subacute Spine Pain: The SPINE CARE Randomized Clinical Trial. JAMA. 2022Dec 20;328(23):2334–44. 10.1001/jama.2022.22625. 36538309 10.1001/jama.2022.22625 PMC9856689

[72] Hansson EE, Jönsson-Lundgren M, Ronnheden AM, Sörensson E, Bjärnung Å, Dahlberg LE. Effect of an education programme for patients with osteoarthritis in primary care - A randomized controlled trial. BMC Musculoskelet Disord. 2010;11. 10.1186/1471-2474-11-244 10.1186/1471-2474-11-244 PMC2987970 20969809

[73] Traeger AC, Lee H, Hübscher M, Skinner IW, Moseley GL, Nicholas MK, et al. Effect of Intensive Patient Education vs Placebo Patient Education on Outcomes in Patients with Acute Low Back Pain: A Randomized Clinical Trial. JAMA Neurol. 2019Feb 1;76(2):161–9. 10.1001/jamaneurol.2018.3376. 30398542 10.1001/jamaneurol.2018.3376 PMC6440280

[74] Mecklenburg G, Smittenaar P, Erhart-Hledik JC, Perez DA, Hunter S. Effects of a 12-week digital care program for chronic knee pain on pain, mobility, and surgery risk: Randomized controlled trial. J Med Internet Res. 2018 Apr 1;20(4). 10.2196/jmir.9667 10.2196/jmir.9667 PMC5943627 29695370

[75] Calner T, Nordin C, Eriksson MK, Nyberg L, Gard G, Michaelson P. Effects of a self-guided, web-based activity programme for patients with persistent musculoskeletal pain in primary healthcare: A randomized controlled trial. Eur J Pain (United Kingdom). 2017Jul 1;21(6):1110–20. 10.1002/ejp.1012. 10.1002/ejp.1012 28464364

[76] Semrau J, Hentschke C, Peters S, Pfeifer K. Effects of behavioural exercise therapy on the effectiveness of multidisciplinary rehabilitation for chronic non-specific low back pain: a randomised controlled trial. BMC Musculoskelet Disord. 2021 Dec 1;22(1). 10.1186/s12891-021-04353-y 10.1186/s12891-021-04353-y PMC8164753 34051780

[77] Andersen LN, Juul Kristensen B, Sorensen TL, Herborg LG, Roessler KK, Sogaard K. Efficacy of Tailored Physical Activity or Chronic Pain Self-Management Programme on return to work for sick-listed citizens: A 3-month randomised controlled trial. Scand J Public Health. 2015;43(7):694–703. 10.1177/1403494815591687. 26113171 10.1177/1403494815591687

[78] Løchting I, Storheim K, Werner EL, Småstuen Cvancarova M, Grotle M. Evaluation of individualized quality of life and illness perceptions in low back pain. A patient education cluster randomized controlled trial. Patient Educ Couns. 2016 Dec 1;99(12):1992–8. 10.1016/j.pec.2016.05.015 10.1016/j.pec.2016.05.015 27486051

[79] Bair MJ, Ang D, Wu J, Outcalt SD, Sargent C, Kempf C, et al. Evaluation of Stepped Care for Chronic Pain (ESCAPE) in veterans of the iraq and afghanistan conflicts a randomized clinical trial. JAMA Intern Med. 2015May 1;175(5):682–9. 10.1001/jamainternmed.2015.97. 25751701 10.1001/jamainternmed.2015.97

[80] Walsh N, Jones L, Phillips S, Thomas R, Odondi L, Palmer S, et al. Facilitating Activity and Self-management for people with Arthritic knee, hip or lower back pain (FASA): A cluster randomised controlled trial. Musculoskelet Sci Pract. 2020Dec;1:50. 10.1016/j.msksp.2020.102271. 10.1016/j.msksp.2020.102271 33068901

[81] Mansell G, Storheim K, Løchting I, Werner EL, Grotle M. Identification of Indirect Effects in a Cognitive Patient Education (COPE) Intervention for Low Back Pain. Vol. 97, Original Research 1138 Physical Therapy. 2017. 10.1093/ptj/pzx091 10.1093/ptj/pzx091 PMC5803786 29186635

[82] Andersen LN, Juul-Kristensen B, Sørensen TL, Herborg LG, Roessler KK, Søgaard K. Longer term follow-up of the effects of tailored physical activity or chronic pain self-management Programme on return-To-work: A randomized controlled trial. J Rehabil Med. 2016Nov 1;48(10):887–92. 10.2340/16501977-2159. 27786344 10.2340/16501977-2159

[83] Galan-Martin MA, Montero-Cuadrado F, Lluch-Girbes E, Coca-López MC, Mayo-Iscar A, Cuesta-Vargas A. Pain neuroscience education and physical therapeutic exercise for patients with chronic spinal pain in spanish physiotherapy primary care: A pragmatic randomized controlled trial. J Clin Med. 2020 Apr 1;9(4). 10.3390/jcm9041201 10.3390/jcm9041201 PMC7230486 32331323

[84] Van Oosterwijck J Van, Meeus M, Paul L, De Schryver M, Pascal A, Lambrecht L, et al. Pain Physiology Education Improves Health Status and Endogenous Pain Inhibition in Fibromyalgia A Double-Blind Randomized Controlled Trial [Internet]. 2013. 10.1097/AJP.0b013e31827c7a7d 10.1097/AJP.0b013e31827c7a7d 23370076

[85] Linden M, Scherbe S, Cicholas B. Randomized controlled trial on the effectiveness of cognitive behavior group therapy in chronic back pain patients. J Back Musculoskelet Rehabil. 2014;27(4):563–8. 10.3233/BMR-140518. 25096315 10.3233/BMR-140518

[86] López-López L, Ariza-Mateos MJ, Rodríguez-Torres J, Cabrera-Martos I, Granados-Santiago M, Torres-Sánchez I, et al. Results of a self-management program added to standard physical therapy in chronic neck pain. Patient Educ Couns. 2021Jun 1;104(6):1438–44. 10.1016/j.pec.2020.11.014. 33246873 10.1016/j.pec.2020.11.014

[87] Gustavsson C, Denison E, Von Koch L. Self-management of persistent neck pain: Two-year follow-up of a randomized controlled trial of a multicomponent group intervention in primary health care. Spine (Phila Pa 1976). 2011 Dec 1;36(25):2105–15. 10.1097/BRS.0b013e3182028b04 10.1097/BRS.0b013e3182028b04 21358487

[88] Gustavsson C, Denison E, von Koch L. Self-management of persistent neck pain: A randomized controlled trial of a multi-component group intervention in primary health care. Eur J Pain. 2010;14(6):630.e1-630.e11. 10.1016/j.ejpain.2009.10.004. 19939717 10.1016/j.ejpain.2009.10.004

[89] Nøst TH, Steinsbekk A, Bratås O, Grønning K. Short-term effect of a chronic pain selfmanagement intervention delivered by an easily accessible primary healthcare service: A randomised controlled trial. BMJ Open. 2018 Dec 1;8(12). 10.1136/bmjopen-2018-023017 10.1136/bmjopen-2018-023017 PMC6303596 30530580

[90] Elwyn G, Pickles T, Edwards A, Kinsey K, Brain K, Newcombe RG, et al. Supporting shared decision making using an Option Grid for osteoarthritis of the knee in an interface musculoskeletal clinic: A stepped wedge trial. Patient Educ Couns. 2016Apr 1;99(4):571–7. 10.1016/j.pec.2015.10.011. 26566194 10.1016/j.pec.2015.10.011

[91] Kohns DJ, Urbanik CP, Geisser ME, Schubiner H, Lumley MA. The effects of a pain psychology and neuroscience self-evaluation internet intervention: A randomized controlled trial. Clin J Pain. 2020Sep 1;36(9):683–92. 10.1097/AJP.0000000000000857. 32520816 10.1097/AJP.0000000000000857

[92] Chimenti RL, Post AA, Rio EK, Moseley GL, Dao M, Mosby H, et al. The effects of pain science education plus exercise on pain and function in chronic Achilles tendinopathy: A blinded, placebo-controlled, explanatory, randomized trial. Pain. 2023Jan 1;164(1):E47-65. 10.1097/j.pain.0000000000002720. 36095045 10.1097/j.pain.0000000000002720 PMC10016230

[93] Becker A, Angerer P, Weber J, Müller A. The prevention of musculoskeletal complaints: long-term effect of a work-related psychosocial coaching intervention compared to physiotherapy alone—a randomized controlled trial. Int Arch Occup Environ Health. 2020Oct 1;93(7):877–89. 10.1007/s00420-020-01538-1. 32274576 10.1007/s00420-020-01538-1 PMC7452937

[94] Gustavsson C, von Koch L. Pain self-management intervention supports successful attainment of self-selected rehabilitation goals—secondary analysis of a randomized controlled trial. Heal Expect. 2022Jun 1;25(3):1157–67. 10.1111/hex.13469. 10.1111/hex.13469 PMC9122423 35285115

[95] Murray A, Hall A, Williams GC, McDonough SM, Ntoumanis N, Taylor I, et al. Assessing physiotherapists’ communication skills for promoting patient autonomy for self-management: reliability and validity of the communication evaluation in rehabilitation tool. Disabil Rehabil. 2019Jul 3;41(14):1699–705. 10.1080/09638288.2018.1443159. 29485325 10.1080/09638288.2018.1443159

[96] Louw A, Zimney K, Johnson EA, Kraemer C, Fesler J, Burcham T. De-educate to re-educate: aging and low back pain. Aging Clin Exp Res. 2017Dec 1;29(6):1261–9. 10.1007/s40520-017-0731-x. 28275956 10.1007/s40520-017-0731-x

[97] Martins C, Sayegh S, Faundez A, Fourchet F, Bothorel H. Effectiveness of a Group-Based Rehabilitation Program Combining Education with Multimodal Exercises in the Treatment of Patients with Nonspecific Chronic Low Back Pain: A Retrospective Uncontrolled Study. Biology (Basel). 2022 Oct 1;11(10). 10.3390/biology11101508 10.3390/biology11101508 PMC9598691 36290412

[98] Markus M, Euhus A, Bethge M. Effectiveness of behavioural medical rehabilitation under real-life conditions in Germany: A propensity-score matched analysis. J Rehabil Med Orig Rep J Rehabil Med. 2021;53:0. 10.2340/16501977-jrm.v53.46910.2340/jrm.v53.46934672356

[99] Slater H, Briggs AM, Bunzli S, Davies SJ, Smith AJ, Quintner JL. Engaging consumers living in remote areas of Western Australia in the self-management of back pain: A prospective cohort study. BMC Musculoskelet Disord. 2012;13. 10.1186/1471-2474-13-69 10.1186/1471-2474-13-69 PMC3439262 22578207

[100] Egerton T, Bolton J, Short CE, Bennell KL. Exploring changes, and factors associated with changes, in behavioural determinants from a low-cost, scalable education intervention about knee osteoarthritis: An observational cohort study. BMC Musculoskelet Disord. 2021 Dec 1;22(1). 10.1186/s12891-021-04751-2 10.1186/s12891-021-04751-2 PMC8502260 34627203

[101] Johnsen MB, Roos E, Grønne DT, Bråten LCH, Skou ST. Impact of educational level and employment status on short-term and long-term pain relief from supervised exercise therapy and education: An observational study of 22 588 patients with knee and hip osteoarthritis. BMJ Open. 2021 Apr 14;11(4). 10.1136/bmjopen-2020-045156 10.1136/bmjopen-2020-045156 PMC8054081 33853803

[102] Carr JL, Moffett JAK, Sharp DM, Haines DR. Is the Pain Stages of Change Questionnaire (PSOCQ) a useful tool for predicting participation in a self-management programme? Further evidence of validity, on a sample of UK pain clinic patients. BMC Musculoskelet Disord. 2006Dec;14:7. 10.1186/1471-2474-7-101. 10.1186/1471-2474-7-101 PMC1769371 17169141

[103] Coutu MF, Légaré F, Stacey D, Durand MJ, Corbière M, Bainbridge L, et al. Occupational therapists’ shared decision-making behaviors with patients having persistent pain in a work rehabilitation context: A cross-sectional study. Patient Educ Couns. 2015Jul 1;98(7):864–70. 10.1016/j.pec.2015.03.015. 25850756 10.1016/j.pec.2015.03.015

[104] Davies S, Quintner J, Parsons R, Parkitny L, Knight P, Forrester E, et al. Preclinic Group Education Sessions Reduce Waiting Times and Costs at Public Pain Medicine Units. Pain Med. 2011 Jan 1;12(1):59–71. 10.1111/J.1526-4637.2010.01001.X. Cited 2024 May 2 10.1111/j.1526-4637.2010.01001.x 21087401

[105] Banerjee A, Hendrick P, Blake H. Predictors of self-management in patients with chronic low back pain: a longitudinal study. BMC Musculoskelet Disord. 2022 Dec 1;23(1). 10.1186/s12891-022-05933-2 10.1186/s12891-022-05933-2 PMC9727914 36476492

[106] Chen CH, Chuang HY, Lee Y, Elwyn G, Hou WH, Kuo KN. Relationships among Antecedents, Processes, and Outcomes for Shared Decision Making: A Cross-Sectional Survey of Patients with Lumbar Degenerative Disease. Med Decis Mak. 2022Apr 1;42(3):352–63. 10.1177/0272989X211024980. 10.1177/0272989X211024980 34634947

[107] Burns JW, Glenn B, Lofland K, Bruehl S, Harden RN. Stages of change in readiness to adopt a self-management approach to chronic pain: The moderating role of early-treatment stage progression in predicting outcome. Pain. 2005Jun;115(3):322–31. 10.1016/j.pain.2005.03.007. 15911159 10.1016/j.pain.2005.03.007

[108] Clare A, MacNeil S, Bunton T, Jarrett S. ‘The Doctor doesn’t need to see you now’: reduction in general practice appointments following group pain management. Br J Pain. 2019May 1;13(2):121–9. 10.1177/2049463718812501. 31019694 10.1177/2049463718812501 PMC6463353

[109] Grevnerts HT, Krevers B, Kvist J. Treatment decision-making process after an anterior cruciate ligament injury: patients’, orthopaedic surgeons’ and physiotherapists’ perspectives. BMC Musculoskelet Disord. 2022 Dec 1;23(1). 10.1186/s12891-022-05745-4 10.1186/s12891-022-05745-4 PMC9380364 35974318

[110] Ohagan ET, Di Pietro F, Traeger AC, Cashin AG, Hodges PW, Wand BM, et al. What messages predict intention to self-manage low back pain? A study of attitudes towards patient education. Pain. 2022Aug 1;163(8):1489–96. 10.1097/j.pain.0000000000002530. 34784310 10.1097/j.pain.0000000000002530

[111] Fry C, Langley-Johnson C, Maksymuk D, Paling C, Marks J, Webb C, et al. Activate your back: collaborating with council services to encourage people with low back pain to self-manage in community settings.; 10.1016/j.physio.2018.11.291. Cited 2024 May 23

[112] Rawlinson G, Connell L, Tarling R, Beaver K. An exploration of adherence to self-management physiotherapy programmes in musculoskeletal physiotherapy using the COM B model and TDF framework. Physiotherapy. 2021Dec;113:e9-10. 10.1016/j.physio.2021.10.233.

[113] Wanless B, Bermingham C, Yeates N, O’Rahelly J. Creating a valuable patient information resource for MSK conditions: By not re-inventing the wheel. Physiotherapy. 2021Dec;113:e120–1. 10.1016/j.physio.2021.10.106.

[114] Buchan S. How effective is a local musculoskeletal condition specific management website? A retrospective evaluation of practice Physiotherapy. 2022Feb;114: e92. 10.1016/j.physio.2021.12.035.

[115] Roberts S, Busby E. Implementing clinical guidelines into practice: The Osteoarthritis Self-management and Independent-living Support (OASIS) group—A service evaluation. Musculoskeletal Care. 2020Sep 1;18(3):404–11. 10.1002/msc.1471. 32253817 10.1002/msc.1471

[116] Castle C. MSK self management smart phone app in general practice. Physiotherapy. 2020;107(1):e120. Cited 2024 Jun 25

[117] Williams D, Bewley A, Varsani P, Pankhania S, Liasides C, Cobb E, et al. Piloting an innovative model for stakeholder engagement within residential rehabilitation at a national specialist centre. Physiotherapy [Internet]. 2019Jan;105:e148–9. 10.1016/j.physio.2018.11.144.

[118] Dando M, Grenfell J, Morgan J, Jones E, Knight-Davis M, Letchford R. Shared Decision Making training improves confidence in clinicians to facilitate collaborative decisions in musculoskeletal physiotherapy. Physiotherapy [Internet]. November 2019;2021(113):e153–4. 10.1016/j.physio.2021.10.152.

[119] Hutting N, Richardson J, Johnston V. The role of self-management in the treatment of musculoskeletal disorders. Man Ther. 2016Sep;25:e15–7. 10.1016/j.math.2016.05.013.

[120] Parish A, Ashton J. The use of Patient Reported Outcome Measures with patients in a musculoskeletal outpatient physiotherapy setting to enhance Shared Decision Making. Physiotherapy. 2021Dec;113:e104–5. 10.1016/j.physio.2021.10.083.

[121] Walker A, Mcclellan C, Sheldon H, Wanless B, Berry A. Using the NICE evidence standards for digital health technologies to evaluate a digital self-management tool for people with musculoskeletal conditions. Physiother (United Kingdom). 2021;114S1:e111. 10.1016/j.physio.2021.12.060

[122] Pattern H, Stewart C, Horler C, Hemmings S, Daluiso G. Using health coaching and the PatientActivation Measure® to supportself-management within musculoskeletaloutpatients: A service improvementproject. Virtual Physiother UK. 2021;114(1):71–244. 10.1016/j.physio.2021.12.128. Cited 2024 May 2

[123] Gustavsson C, Nordlander J, Söderlund A. Activity and life-role targeting rehabilitation for persistent pain: feasibility of an intervention in primary healthcare. Eur J Physiother. 2018Jul 3;20(3):141–51. 10.1080/21679169.2018.1426784.

[124] Thorstensson CA, Garellick G, Rystedt H, Dahlberg LE. Better Management of Patients with Osteoarthritis: Development and Nationwide Implementation of an Evidence-Based Supported Osteoarthritis Self-Management Programme. Musculoskeletal Care. 2015Jun 1;13(2):67–75. 10.1002/msc.1085. 25345913 10.1002/msc.1085

[125] Fioratti I, Miyamoto GC, Fandim V, Ribeiro CPP, Batista GD, Freitas GE, et al. Feasibility, Usability, and Implementation Context of an Internet-Based Pain Education and Exercise Program for Chronic Musculoskeletal Pain: Pilot Trial of the ReabilitaDOR Program. JMIR Form Res. 2022 Aug 1;6(8). 10.2196/35743 10.2196/35743 PMC9472033 35776863

[126] Perry J, VanDenKerkhof EG, Wilson R, Tripp DA. Guided Internet-based Psycho-educational Intervention Using Cognitive Behavioral Therapy and Self-management for Individuals with Chronic Pain: A Feasibility Study. Pain Manag Nurs. 2017Jun 1;18(3):179–89. 10.1016/j.pmn.2016.12.003. 28433488 10.1016/j.pmn.2016.12.003

[127] Schütze R, Slater H, O’Sullivan P, Thornton J, Finlay-Jones A, Rees CS. Mindfulness-based functional therapy: A preliminary open trial of an integrated model of care for people with persistent low back pain. Front Psychol. 2014;5(AUG). 10.3389/fpsyg.2014.00839 10.3389/fpsyg.2014.00839 PMC4120853 25136324

[128] Anderson AM, Lavender EC, Dusabe-Richards E, Mebrahtu TF, McGowan L, Conaghan PG, et al. Peer mentorship to improve self-management of hip and knee osteoarthritis: A randomised feasibility trial. BMJ Open. 2021 Jul 21;11(7). 10.1136/bmjopen-2020-045389 10.1136/bmjopen-2020-045389 PMC8296761 34290063

[129] Sharma S, Jensen MP, Moseley GL, Abbott JH. Results of a feasibility randomised clinical trial on pain education for low back pain in Nepal: The Pain Education in Nepal-Low Back Pain (PEN-LBP) feasibility trial. BMJ Open. 2019 Mar 1;9(3). 10.1136/bmjopen-2018-026874 10.1136/bmjopen-2018-026874 PMC6475174 30918037

[130] Rufa A, Beissner K, Dolphin M. The use of pain neuroscience education in older adults with chronic back and/or lower extremity pain. Physiother Theory Pract. 2019Jul 3;35(7):603–13. 10.1080/09593985.2018.1456586. 29601227 10.1080/09593985.2018.1456586

[131] Smith BE, Hendrick P, Bateman M, Moffatt F, Rathleff MS, Selfe J, et al. A loaded self-managed exercise programme for patellofemoral pain: A mixed methods feasibility study. BMC Musculoskelet Disord. 2019 Mar 27;20(1). 10.1186/s12891-019-2516-1 10.1186/s12891-019-2516-1 PMC6438027 30917806

[132] Yin Z, Li S, Ortega C, Bobadilla R, Winkler PL, Hernández AE, et al. Impacts on patient-centered outcomes of a chronic pain self-management program in a rural community: A feasibility study. Geriatr Nurs (Minneap). 2021Sep 1;42(5):1198–203. 10.1016/j.gerinurse.2021.06.026. 10.1016/j.gerinurse.2021.06.026 34425422

[133] Monaghan J, Adams N, Fothergill M. An evaluation of a pain education programme for physiotherapists in clinical practice. Musculoskeletal Care. 2018Mar 1;16(1):103–11. 10.1002/msc.1218. 29076620 10.1002/msc.1218

[134] Johnston V, Strong J, Gargett S, Jull G, Ellis N, Johnston V. Enhancing the vocational outcomes of people with chronic disabilities caused by a musculoskeletal condition: Development and evaluation of content of self-management training modules. Work. 2014:9(3):55-464. 10.3233/WOR-131722. 10.3233/WOR-131722 24004780

[135] Hutting N, Detaille SI, Heerkens YF, Engels JA, Staal JB, Nijhuis-van der Sanden MWG. Experiences of Participants in a Self-Management Program for Employees with Complaints of the Arm, Neck or Shoulder (CANS): A Mixed Methods Study. J Occup Rehabil. 2017 Mar 1;27(1):35–48. 10.1007/s10926-016-9630-9. 10.1007/s10926-016-9630-9 PMC5306216 26875155

[136] Parchment A, Lawrence W, Rahman E, Townsend N, Wainwright E, Wainwright D. How useful is the Making Every Contact Count Healthy Conversation Skills approach for supporting people with musculoskeletal conditions? J Public Heal. 2022Oct 1;30(10):2389–405. 10.1007/s10389-022-01718-y. 10.1007/s10389-022-01718-y PMC9067897 35530417

[137] Sheppard DM, Gargett S, MacKenzie A, Jull G, Johnston V, Strong J, et al. Implementing a Self-Management Intervention for People with a Chronic Compensable Musculoskeletal Injury in a Workers Compensation Context: A Process Evaluation. J Occup Rehabil. 2015Jun 22;25(2):412–22. 10.1007/s10926-014-9551-4. 25385198 10.1007/s10926-014-9551-4

[138] Button K, Nicholas K, Busse M, Collins M, Spasić I. Integrating self-management support for knee injuries into routine clinical practice: TRAK intervention design and delivery. Musculoskelet Sci Pract. 2018Feb;1(33):53–60. 10.1016/j.msksp.2017.11.002. 10.1016/j.msksp.2017.11.002 29172113

[139] Coutu MF, Légaré F, Durand MJ, Corbière M, Stacey D, Bainbridge L, et al. Operationalizing a Shared Decision Making Model for Work Rehabilitation Programs: A Consensus Process. J Occup Rehabil. 2015Mar 1;25(1):141–52. 10.1007/s10926-014-9532-7. 25001070 10.1007/s10926-014-9532-7

[140] Darlow B, Brown M, Grainger R, Hudson B, Briggs AM, Haxby Abbott J, et al. Stakeholder views about a novel consumer health resource for knee osteoarthritis. Osteoarthr Cartil Open. 2020 Jun 1;2(2). 10.1016/j.ocarto.2020.100058 10.1016/j.ocarto.2020.100058 PMC9718132 36474583

[141] Farin E, Nagl M, Ullrich A. The comprehensibility of health education programs: Questionnaire development and results in patients with chronic musculoskeletal diseases. Patient Educ Couns. 2013Feb;90(2):239–46. 10.1016/j.pec.2012.10.004. 23127897 10.1016/j.pec.2012.10.004

[142] Matthias MS, Mcguire AB, Kukla M, Daggy J, Myers LJ, Bair MJ. A Brief Peer Support Intervention for Veterans with Chronic Musculoskeletal Pain: A Pilot Study of Feasibility and Effectiveness. Pain Med. 2015 Jan 1;16(1):81. 10.1111/PME.12571. Cited 2024 May 2 10.1111/pme.12571 PMC4793916 25312858

[143] Janevic M, Robinson-Lane SG, Murphy SL, Courser R, Piette JD. A Pilot Study of a Chronic Pain Self-Management Program Delivered by Community Health Workers to Underserved African American Older Adults. 2022Dec 1;23(12):1965–78. 10.1093/pm/pnaa468. 10.1093/pm/pnaa468 PMC9714529 33779759

[144] Carpenter KM, Stoner SA, Mundt JM, Stoelbc B. An online self-help CBT intervention for chronic lower back pain. Clin J Pain. 2012;28(1):14–22. 10.1097/AJP.0b013e31822363db. 21681084 10.1097/AJP.0b013e31822363db PMC3184315

[145] Amorim AB, Pappas E, Simic M, Ferreira ML, Jennings M, Tiedemann A, et al. Integrating Mobile-health, health coaching, and physical activity to reduce the burden of chronic low back pain trial (IMPACT): A pilot randomised controlled trial. BMC Musculoskelet Disord. 2019 Feb 11;20(1). 10.1186/s12891-019-2454-y 10.1186/s12891-019-2454-y PMC6371593 30744606

[146] van Oosterwijck J, Nijs J, Meeus M, Truijen S, Craps J, van den Keybus N, et al. Pain neurophysiology education improves cognitions, pain thresholds, and movement performance in people with chronic whiplash: A pilot study. J Rehabil Res Dev. 2011;48(1):43–58. 10.1682/JRRD.2009.12.0206. 21328162 10.1682/jrrd.2009.12.0206

[147] Patel S, Ngunjiri A, Hee SW, Yang Y, Brown S, Friede T, et al. Primum non nocere: Shared informed decision making in low back pain - A pilot cluster randomised trial. BMC Musculoskelet Disord. 2014 Aug 21;15(1). 10.1186/1471-2474-15-282 10.1186/1471-2474-15-282 PMC4247192 25146587

[148] Brand CA, Amatya B, Gordon B, Tosti T, Gorelik A. Redesigning care for chronic conditions: Improving hospital-based ambulatory care for people with osteoarthritis of the hip and knee. Intern Med J. 2010Jun;40(6):427–36. 10.1111/j.1445-5994.2009.01960.x. 19323698 10.1111/j.1445-5994.2009.01960.x

[149] Brady B, Sidhu B, Jennings M, Boland R, Hassett G, Chipchase L, et al. The feasibility of implementing a cultural mentoring program alongside pain management and physical rehabilitation for chronic musculoskeletal conditions: results of a controlled before-and-after pilot study. BMC Musculoskelet Disord. 2023 Dec 1;24(1). 10.1186/s12891-022-06122-x 10.1186/s12891-022-06122-x PMC9850562 36658511

[150] Miller J, MacDermid JC, Richardson J, Walton DM, Gross A. Depicting individual responses to physical therapist led chronic pain self-management support with pain science education and exercise in primary health care: multiple case studies. Arch Physiother. 2017 Dec 1;7(1). 10.1186/s40945-017-0032-x 10.1186/s40945-017-0032-x PMC5759926 29340199

[151] Casey MB, Cotter N, Kelly C, Mc Elchar L, Dunne C, Neary R, et al. Exercise and Acceptance and Commitment Therapy for Chronic Pain: A Case Series with One-Year Follow-Up. Musculoskeletal Care. 2020 Mar 1;18(1):64–73. 10.1002/MSC.1444. Cited 2024 May 2 10.1002/msc.1444 31967395

[152] Caneiro JP, Smith A, Rabey M, Moseley GL, O’Sullivan P. Process of change in pain-related fear: Clinical insights from a single case report of persistent back pain managed with cognitive functional therapy. J Orthop Sports Phys Ther. 2017Sep 1;47(9):637–51. 10.2519/jospt.2017.7371. 28704623 10.2519/jospt.2017.7371

[153] Walston Z, Nmonag P, Spiker L, Yake D. Use of cognitive behavioural therapy with usual physical therapy intervention for individuals who are unemployed secondary to chronic low back pain: A case series. Musculoskeletal Care. 2019Sep 1;17(3):269–73. 10.1002/msc.1422. 31373424 10.1002/msc.1422

[154] Zimney K, Louw A, Puentedura EJ. Use of Therapeutic Neuroscience Education to address psychosocial factors associated with acute low back pain: A case report. Physiother Theory Pract. 2014Apr;30(3):202–9. 10.3109/09593985.2013.856508. 24252071 10.3109/09593985.2013.856508

[155] Iles RA, Taylor NF, Davidson M, O’Halloran P. An effective coaching intervention for people with low recovery expectations and low back pain: A content analysis. J Back Musculoskelet Rehabil. 2014;27(1):93–101. 10.3233/BMR-130424. 23948852 10.3233/BMR-130424

[156] Alamam D, Alhowimel A, Alodaibi F, Alsobayel H. Are healthcare providers offering the proper education for people with low back pain? Content analysis of educational materials. J Back Musculoskelet Rehabil. 2022;35(6):1269–76. 10.3233/BMR-210232. 35599466 10.3233/BMR-210232

[157] Hurley DA, Keogh A, Ardle DM, Hall AM, Richmond H, Guerin S, et al. Evaluation of an e-learning training program to support implementation of a group-based, theory-driven, self-management intervention for osteoarthritis and low-back pain: Pre-post study. J Med Internet Res. 2019 Mar 1;21(3). 10.2196/11123 10.2196/11123 PMC6427104 30843863

[158] Wang X, Urban H, Bennell KL, Dickson C, Dobson F, Fransen M, et al. My joint pain, a web-based resource, effects on education and quality of care at 24 months. BMC Musculoskelet Disord. 2020 Feb 6;21(1). 10.1186/s12891-020-3074-2 10.1186/s12891-020-3074-2 PMC7006132 32028927

[159] Hutting N, Detaille SI, Engels JA, Heerkens YF, Staal JB, Nijhuis-van der Sanden MWG. Development of a self-management program for employees with complaints of the arm, neck, and/or shoulder: An intervention mapping approach. J Multidiscip Healthc. 2015 Jul 1;8:307–20. 10.2147/JMDH.S82809 10.2147/JMDH.S82809 PMC4492641 26170689

[160] Fritsch CG, Ferreira PH, Prior JL, Vesentini G, Schlotfeldt P, Eyles J, et al. TEXT4myBACK – The Development Process of a Self-Management Intervention Delivered Via Text Message for Low Back Pain. Arch Rehabil Res Clin Transl. 2021Jun;3(2): 100128. 10.1016/j.arrct.2021.100128. 34179764 10.1016/j.arrct.2021.100128 PMC8212000

[161] Lin I, Wiles L, Waller R, Caneiro JP, Nagree Y, Straker L, et al. Patient-centred care: The cornerstone for high-value musculoskeletal pain management. Vol. 54, British Journal of Sports Medicine. BMJ Publishing Group; 2020. p. 1240–2. 10.1136/bjsports-2019-101918 10.1136/bjsports-2019-101918 32586944

[162] Buchbinder R, van Tulder M, Öberg B, Costa LM, Woolf A, Schoene M, et al. Low back pain: a call for action. Lancet. 2018 Jun 9;391(10137):2384–8. 10.1016/S0140-6736(18)30488-4. Cited 2024 Jun 17 10.1016/S0140-6736(18)30488-4 29573871

[163] Hutting N. Supported self-management in musculoskeletal physiotherapy: the time is now!–Guest editorial. Eur J Physiother. 2024; 10.1080/21679169.2024.2337054. Cited 2024 Apr 23

[164] Taylor SJ, Carnes D, Homer K, Pincus T, Kahan BC, Hounsome N, et al. Improving the self-management of chronic pain: COping with persistent Pain, Effectiveness Research in Self-management (COPERS). Program Grants Appl Res. 2016;4(14):1–440. 10.3310/pgfar04140. 27656730

[165] C.R. M, D.T. E, K. B, K. G, K. H, S. M, et al. Rethinking the patient: using Burden of Treatment Theory to understand the changing dynamics of illness. BMC Health Serv Res. 2014;14:281. 10.1186/1472-6963-14-281 PMC4080515 24969758

[166] Hutting N, Caneiro JP, Ong’wen OM, Miciak M, Roberts L. Patient-centered care in musculoskeletal practice: Key elements to support clinicians to focus on the person. Musculoskelet Sci Pract. 2022;57:10243457. 10.1016/j.msksp.2021.102434. 10.1016/j.msksp.2021.102434 34376367

[167] Hutting N, Caneiro JPP, Ong’wen OM, Miciak M, Roberts L. Person-centered care for musculoskeletal pain: Putting principles into practice. Dec 1, 2022 p. 102663. 10.1016/j.msksp.2022.102663 36113362

[168] NHS England » Involving people in their own care. URL:https://www.england.nhs.uk/ourwork/patient-participation/. Cited 2022 Sep 23

[169] Salazar-Méndez J, Cuyul-Vásquez I, Ponce-Fuentes F, Guzmán-Muñoz E, Núñez-Cortés R, Huysmans E, et al. Pain neuroscience education for patients with chronic pain: A scoping review from teaching–learning strategies, educational level, and cultural perspective. Patient Educ Couns. 2024;123(February). 10.1016/j.pec.2024.108201 10.1016/j.pec.2024.108201 38387389

[170] Lorimer Moseley G, Leake HB, Beetsma AJ, Watson JA, Butler DS, van der Mee A, et al. Teaching Patients About Pain: The Emergence of Pain Science Education, its Learning Frameworks and Delivery Strategies. J Pain. 2023;xxx(xxx):1–14. 10.1016/j.jpain.2023.11.008 10.1016/j.jpain.2023.11.008 37984510

[171] Lyng KD, Larsen JB, Birnie KA, Stinson J, Hoegh MS, Palsson TS, et al. Participatory research : a Priority Setting Partnership for chronic musculoskeletal pain in. Scand J Pain [Internet]. 2023;23(2):402–15. 10.1515/sjpain-2022-0019. 35918804 10.1515/sjpain-2022-0019

[172] Caneiro JP, Smith A, Bunzli S, Linton S, Moseley GL, O’Sullivan P. From Fear to Safety: A Roadmap to Recovery From Musculoskeletal Pain. Phys Ther. 2022;102(2):1–12. 10.1093/ptj/pzab271. 10.1093/ptj/pzab271 34971393

[173] Bomhof-Roordink H, Gärtner FR, Stiggelbout AM, Pieterse AH. Key components of shared decision making models: A systematic review. BMJ Open. 2019;9(12). 10.1136/bmjopen-2019-031763 10.1136/bmjopen-2019-031763 PMC6937101 31852700

[174] Wood L, Hendrick PA. A systematic review and meta-analysis of pain neuroscience education for chronic low back pain: Short-and long-term outcomes of pain and disability. Eur J Pain. 2019 Feb 1;23(2):234–49. 10.1002/EJP.1314. Cited 2024 May 8 10.1002/ejp.1314 30178503

[175] Watson JA, Ryan CG, Cooper L, Ellington D, Whittle R, Lavender M, et al. Pain Neuroscience Education for Adults With Chronic Musculoskeletal Pain: A Mixed-Methods Systematic Review and Meta-Analysis. J Pain. 2019Oct 1;20(10):1140.e1-1140.e22. 10.1016/J.JPAIN.2019.02.011. 30831273 10.1016/j.jpain.2019.02.011

[176] Tousignant-Laflamme Y, Christopher S, Clewley D, Ledbetter L, Cook CJCE, Cook CJCE. Does shared decision making results in better health related outcomes for individuals with painful musculoskeletal disorders? A systematic review. J Man Manip Ther [Internet]. 2017;25(3):144–50. 10.1080/10669817.2017.1323607. 28694677 10.1080/10669817.2017.1323607 PMC5498795

[177] Monaghan J, Fothergill M, Adams N. The challenges of self-management of low back pain from the physiotherapist perspective. Physiotherapy. 2016 Nov 1;102:e177. 10.1016/j.physio.2016.10.211. Cited 2024 Jun 17

[178] Moore CL, Kaplan SL. A framework and resources for shared decision making: Opportunities for improved physical therapy outcomes. Phys Ther. 2018;98(12):1022–36. 10.1093/PTJ/PZY095. 30452721 10.1093/ptj/pzy095

[179] Jones LE, Roberts LC, Little PS, Mullee MA, Cleland JA, Cooper C. Shared decision-making in back pain consultations: An illusion or reality? Eur Spine J. 2014;23(SUPPL. 1). 10.1007/S00586-014-3187-0 10.1007/s00586-014-3187-0 PMC3946094 24477377

[180] Dierckx K, Deveugele M, Roosen P, Devisch I. Implementation of Shared Decision Making in Physical Therapy: Observed Level of Involvement and Patient Preference. Phys Ther. 2013 Oct 1;93(10):1321–30. 10.2522/PTJ.20120286. Cited 2023 Nov 13 10.2522/ptj.20120286 23641024

[181] Mankelow J, Ryan CG, Green PW, Taylor PC, Martin D. An exploration of primary care healthcare professionals’ understanding of pain and pain management following a brief pain science education. BMC Med Educ [Internet]. 2022;22(1):1–8. 10.1186/s12909-022-03265-2. 35351106 10.1186/s12909-022-03265-2 PMC8962069

[182] van der Gaag M, Heijmans M, Spoiala C, Rademakers J. The importance of health literacy for self-management: A scoping review of reviews. Chronic Illn. 2022;18(2):234–54. 10.1177/17423953211035472. 34402309 10.1177/17423953211035472

[183] Edward J, Carreon LY, Williams MV, Glassman S, Li J. The importance and impact of patients’ health literacy on low back pain management: a systematic review of literature. Spine J [Internet]. 2018;18(2):370–6. 10.1016/j.spinee.2017.09.005. 28939167 10.1016/j.spinee.2017.09.005

[184] Wittink H, Oosterhaven J. Patient education and health literacy. Musculoskelet Sci Pract. 2018Dec;1(38):120–7. 10.1016/j.msksp.2018.06.004. 10.1016/j.msksp.2018.06.004 30017902

[185] Narayanan AS, Stoll KE, Pratson LF, Lin FC, Olcott CW, Del Gaizo DJ. Musculoskeletal Health Literacy is Associated With Outcome and Satisfaction of Total Knee Arthroplasty. J Arthroplasty. 2021Jul 1;36(7):S192–7. 10.1016/J.ARTH.2021.02.075. 33812715 10.1016/j.arth.2021.02.075

[186] Spink A, Wagner I, Orrock P. Common reported barriers and facilitators for self-management in adults with chronic musculoskeletal pain: A systematic review of qualitative studies. Musculoskelet Sci Pract [Internet]. 2021;56(July): 102433. 10.1016/j.msksp.2021.102433. 34416557 10.1016/j.msksp.2021.102433

[187] Artus M, Campbell P, Mallen CD, Dunn KM, Van Der Windt DAW. Generic prognostic factors for musculoskeletal pain in primary care: a systematic review. BMJ Open. 2017 Jan 1;7(1). 10.1136/BMJOPEN-2016-012901. Cited 2024 Apr 26 10.1136/bmjopen-2016-012901 PMC5253570 28096253

[188] Vargas-Prada S, Coggon D. Psychological and psychosocial determinants of musculoskeletal pain and associated disability. Best Pract Res Clin Rheumatol. 2015Jun 1;29(3):374–90. 10.1016/J.BERH.2015.03.003. 26612236 10.1016/j.berh.2015.03.003 PMC4668591

[189] Lotze M, Moseley GL, Moseley GL. Theoretical Considerations for Chronic Pain Rehabilitation. Phys Ther. 2015;95(9):1316–20. 10.2522/ptj.20140581. Cited 2024 Apr 26 10.2522/ptj.20140581 25882484

[190] Johnston S, Irving H, Mill K, Rowan MS, Liddy C. The patient’s voice: An exploratory study of the impact of a group self-management support program. BMC Fam Pract [Internet]. 2012;13(1):1. 10.1186/1471-2296-13-65. 22748018 10.1186/1471-2296-13-65 PMC3431243

[191] Thompson J, Gabriel L, Yoward S, Dawson P. The advanced practitioners’ perspective. Exploring the decision-making process between musculoskeletal advanced practitioners and their patients: An interpretive phenomenological study. 2021. p. 128–36. 10.1002/msc.1562 10.1002/msc.1562 33993603

[192] Zadro J, O’Keeffe M, Maher C. Do physical therapists follow evidence-based guidelines when managing musculoskeletal conditions? Systematic review. BMJ Open. 2019 Oct 1;9(10):e032329. 10.1136/BMJOPEN-2019-032329. Cited 2022 Apr 29 10.1136/bmjopen-2019-032329 PMC6797428 31591090

[193] Charles A, Ham C, Baird B, Alderwick H, Bennet L. Reimagining community services: making the most of our assets. King’s Fund. 2018;(January):118.

[194] Kleiner MJ, Kinsella EA, Miciak M, Teachman G, McCabe E, Walton DM. An integrative review of the qualities of a ‘good’ physiotherapist. Physiother Theory Pract. 2023;39(1):89–116. 10.1080/09593985.2021.1999354. 34881685 10.1080/09593985.2021.1999354

[195] Søndenå P, Dalusio-King G, Hebron C. Conceptualisation of the therapeutic alliance in physiotherapy: is it adequate? 2020; 10.1016/j.msksp.2020.102131. Cited 2024 Jan 9 10.1016/j.msksp.2020.102131 32217276

[196] Hutting N, Johnston V, Staal JB, Heerkens YF. Promoting the use of self-management strategies for people with persistent musculoskeletal disorders: The role of physical therapists. Journal of Orthopaedic and Sports Physical Therapy Movement Science Media; Apr 1, 2019 p. 212–5. 10.2519/jospt.2019.0605 30931733

[197] Kebede S. Ask patients “What matters to you?” rather than “What’s the matter?”. BMJ. 2016;354:i4045 10.1136/bmj.i4045. 10.1136/bmj.i4045 27449399

[198] Carnes D, Homer KE, Miles CL, Pincus T, Underwood M, Rahman A, et al. Effective delivery styles and content for self-management interventions for chronic musculoskeletal pain: A systematic literature review. Clin J Pain. 2012;28(4):344–54. 10.1097/AJP.0b013e31822ed2f3. 22001667 10.1097/AJP.0b013e31822ed2f3

[199] Kearns R, Harris-Roxas B, McDonald J, Song HJ, Dennis S, Harris M. Implementing the Patient Activation Measure (PAM) in clinical settings for patients with chronic conditions: a scoping reviewImplementing the Patient Activation Measure (PAM) in clinical settings for patients with chronic conditions: a scoping review. Integr Healthc J. 2020 Jul 12;22(11). 10.1136/IHJ-2019-000032 10.1136/ihj-2019-000032 PMC10327461 37441314

[200] Hoffmann T, Bakhit M, Michaleff Z. Shared decision making and physical therapy: What, when, how, and why? Brazilian J Phys Ther. 2022;26:100382. 10.1016/j.bjpt.2021.100382. Cited 2024 Jan 10 10.1016/j.bjpt.2021.100382 PMC8784295 35063699

[201] Johnson L, Kirk H, Clark B, Heath S, Royse C, Adams C, et al. Improving personalised care, through the development of a service evaluation tool to assess, understand and monitor delivery. BMJ Open Qual. 2023;12(3):1–8. 10.1136/bmjoq-2023-002324. 10.1136/bmjoq-2023-002324 PMC10481846 37669810

[202] Killingback C, Thompson M, Chipperfield S, Clark C, Williams J. Physiotherapists’ views on their role in self-management approaches: A qualitative systematic review. Physiother Theory Pract. 2022 Dec 2;38(12):2134–48. 10.1080/09593985.2021.1911011. Cited 2024 Jun 24 10.1080/09593985.2021.1911011 33813990

[203] Alexander L, Cooper K, Peters MD, Tricco AC, Khalil H, Evans C, et al. Large Scoping Reviews: Managing volume and potential chaos in a pool of evidence sources. J Clin Epidemiol. 2024;170: 111343. 10.1016/j.jclinepi.2024.111343. 38582403 10.1016/j.jclinepi.2024.111343

[204] Goodwin R, Moffatt F, Hendrick P, Stynes S, Bishop A, Logan P. Evaluation of the First Contact Physiotherapy (FCP) model of primary care: a qualitative insight. Physiotherapy. 2021 Dec 1;113:209. 10.1016/J.PHYSIO.2021.08.003. Cited 2025 Jan 17 10.1016/j.physio.2021.08.003 PMC8612276 34583834

[205] Blackburn J, Hallam F, McComiskie E, Rankin G. Musculoskeletal physiotherapy service standards. Chart Soc Physiother. 2021;(November):1–71.

[206] England NHS. Working in Partnership with People and Communities. 2022. https://www.england.nhs.uk/wpcontent/uploads/2023/05/B1762-guidance-on-working-in-partnership-with-people-and-communities-2.pdf.

[207] taking charge • taking responsibility taking charge • taking responsibility Wigan Borough Clinical Commissioning Group. https://www.wigan.gov.uk/Docs/PDF/Council/Strategies-Plans-and-Policies/Health-Devolution.pdf.

[208] Abel J, Kingston H, Scally A, Hartnoll J, Hannam G, Thomson-Moore A, et al. Reducing emergency hospital admissions: a population health complex intervention of an enhanced model of primary care and compassionate communities. Br J Gen Pract. 2018;68(676):e803–10. 10.3399/BJGP18X699437. 10.3399/bjgp18X699437 PMC6193793 30297434

[209] Forder J, Jones K, Glendinning C, Caiels J, Welch E, Baxter K, et al. Evaluation of the personal health budget pilot programme: executive summary. Discuss Pap 2840_2. 2012;1–4.

[210] Bickerdike L, Booth A, Wilson PM, Farley K, Wright K. Social prescribing: Less rhetoric and more reality. A systematic review of the evidence. BMJ Open. 2017;7(4):e013384. 10.1136/bmjopen-2016-013384. 10.1136/bmjopen-2016-013384 PMC5558801 28389486

[211] Bal MI, Sattoe JNT, Roelofs PDDM, Bal R, van Staa AL, Miedema HS. Exploring effectiveness and effective components of self-management interventions for young people with chronic physical conditions: A systematic review. Patient Educ Couns. 2016Aug 1;99(8):1293–309. 10.1016/J.PEC.2016.02.012. 26954345 10.1016/j.pec.2016.02.012

[212] Wood S, Finnis A, Khan H, Ejbye J. At the heart of health. Realising the value of people and communities Report. Realis value. 2016;(March):55.

[213] Haynes A, Sherrington C, Ramsay E, Kirkham C, Manning S, Wallbank G, et al. “Sharing Success with Someone”: Building therapeutic alliance in physiotherapist-delivered physical activity coaching for healthy aging. Physiother Theory Pract. 2022;38(13):2771–87. 34324406 10.1080/09593985.2021.1946872

[214] Rethorn Z, Rethorn ZD, Pettitt CD. What Is the Effect of Health Coaching Delivered by Physical Therapists? A Systematic Review of Randomized Controlled Trials. Phys Ther. 2019;99:1354–70. 10.1093/ptj/pzz098. Cited 2024 Apr 24 10.1093/ptj/pzz098 31309976

[215] Matthias MS, Hirsh AT, Ofner S, Daggy J. Exploring the Relationships Among Social Support, Patient Activation, and Pain-Related Outcomes. Pain Med (United States). 2022;23(4):676–85. 10.1093/pm/pnab306. 10.1093/pm/pnab306 34718764

[216] England NHS. Supported self-management: peer support guide. 2023;1–12. https://www.wigan.gov.uk/Docs/PDF/Council/Strategies-Plans-and-Policies/Health-Devolution.pdf.

