# Supplementary material for: What does collaborative healthcare for people with musculoskeletal-related conditions look like? A scoping review
Source: BMC Musculoskelet Disord. 2025 Jul 4;26:602. doi: 10.1186/s12891-025-08814-6 (PMC12232000; doi:10.1186/s12891-025-08814-6)
Supplement: Supplementary file 1 — Supplementary Material 1 [file 12891_2025_8814_MOESM1_ESM.docx]

Supplementary file 1

All eligible items mapped to the population, concept, context pneumonic.

| **Study** | Methods | Population | Concept | Context/Country of origin |
| --- | --- | --- | --- | --- |
| Cridland et al (23) | Qualitative | People with shoulder pain (n=8) | Education | Australia |
| McCluskey et al (24) | Qualitative | Workers with persistent musculoskeletal complaints and their significant others (n=31) | Family/carer involvement | UK and Netherlands / Hospital pain management clinic |
| Acker et al (25) | Qualitative | People with shoulder pain (n=10) | Education (Physiotherapy led) | New Zeland |
| Andersen et al (26) | Qualitative | People with persistent back or upper body pain (n=7) | Self-management support (Physiotherapy led) | Denmark |
| Gustavsson and Koch (65) | RCT | People with neck pain (n=94) | Self-management support (Physiotherapy led) | Sweeden |
| Matthias et al (142) | Pilot study | People with persistent musculoskeletal pain (n=17) and peer coaches (n=9) | Peer support and education | Indianapolis, USA |
| Smith et al (131) | Feasibility | People with knee pain (n=25) | Self-management support (Physiotherapy led) | Secondary care, UK |
| Janevic et al (143) | Pilot study | People with persistent musculoskeletal pain (n=31) | Working with communities (Community health worker led) | USA |
| Bernhardsson et al (12) | Qualitative | People with musculoskeletal pain (n=18) | Shared decision making (Physiotherapy led) | Primary care, Sweeden |
| Franklin et al (27) | Qualitative | People with persistent musculoskeletal pain (n=8) | Self-management support | Hospital based, UK /  Pain management clinic |
| Semedo et al (28) | Qualitative | People with persistent musculoskeletal pain (n=7) | Self-management support (Multi-modal rehabilitation (Doctor, Physiotherapists and nurses) | Primary healthcare, Sweeden |
| Fry et al (111) | Oral presentation abstracts | People with back pain (n=65) | Working with communities (Managers, Physiotherapists, council and health co-ordinators) | UK |
| Gustavsson et al (123) | Feasibility | People with persistent musculoskeletal pain (n=65) | Self-management support (Physiotherapy led) | Primary healthcare, Sweeden |
| Haugli et al (66) | RCT | People with persistent musculoskeletal pain (n=61) | Self-management support (Nurses, physiotherapists and physicians) | Norway |
| Iles et al (155) | Content analysis | People with back pain (n=14) | Health coaching and personalised care planning (Health coaches and Physiotherapists) | Australia |
| Monaghan et al (133) | Mixed methods | Physiotherapists (n-10) | Education (Physiotherapy led) | UK |
| Cheng et al (67) | RCT | People with persistent musculoskeletal pain (n=152) | Self-management support | Hospital and clinic based, China / Pain management programme |
| Rawlinson et al (112) | Oral presentation abstracts | People with persistent musculoskeletal pain (n=11) | Self-management support (Physiotherapy led) | UK |
| Carpenter et al (144) | Pilot | People with persistent back pain (n=164) | Self-management support | USA, Seattle |
| Alamam et al (156) | Content analysis | Seventeen education materials from Physiotherapy clinics and hospitals | Education | Hospital and private clinic based, Saudi Arabia |
| Murray et al (95) | Observational | Twenty four physiotherapists | Self-management support (Physiotherapy led) | Hospital based, Ireland |
| Bair et al (29) | Qualitative | People with persistent musculoskeletal pain and depression (n=18) | Self-management support | Primary care, USA, |
| Thorstensson et al (124) | Feasibility | People with hip and knee osteoarthritis (Across 295 practices) | Self-management support (Physiotherapy led) | Sweeden |
| Malfliet et al (68) | RCT | People with persistent back pain (n=120) | Education (Physiotherapy led) | Belgium |
| Skúladóttir et al (30) | Qualitative | People with persistent musculoskeletal pain (n=11) | Self-management support (Physiotherapists, nurses, psychologists, occupational therapists and social workers) | Iceland |
| Werner et al (69) | RCT | People with persistent back pain (n=81) | Education (General practitioners and Physiotherapists) | Primary care, Norway |
| Wanless et al (113) | Oral presentation abstracts | 12 musculoskeletal resources | Education (Physiotherapy led) | UK |
| Louw et al (96) | Observational | People with persistent back pain (n=50) | Education (Physiotherapy led) | USA |
| Miller et al (150) | Case studies | People with persistent musculoskeletal pain (n=6) | Self-management support (Physiotherapy led) | Community health centre, Canada |
| Dragesund and Øien (31) | Qualitative | People with persistent musculoskeletal pain (n=11) | Self-management support (Physiotherapy led) | Norway |
| Moe et al (32) | Qualitative | People with hand, hip or knee osteoarthritis (n=12) | Self-management support (Occupational therapy, Pharmacist, Physiotherapist and Rheumatologist) | Norway - Outpatient clinic / multi-disciplinary clinics |
| Hutting et al (159) | Intervention development | People with musculoskeletal pain (n=15), experts (n=17) | Self-management support | Holland |
| Hutting et al (70) | RCT | People with persistent musculoskeletal pain (n=123) | Self-management support (Physiotherapy led) | Holland |
| Choudhry et al (71) | RCT | People with persistent spinal pain (n=829) | Self-management support (Physiotherapy led) | Primary care clinics, USA |
| Hansson et al (72) | RCT | People with persistent musculoskeletal pain (n=114) | Education (Physiotherapist, orthopaedic surgeon and Occupational therapists) | Sweeden |
| Traeger et al (73) | RCT | People with acute low back pain (n=202) | Education (Physiotherapy led) | Australia |
| Martins et al (97) | Observational | People with persistent low back pain (n=23) | Self-management support (Physiotherapy led) | Switzerland |
| Markus et al (98) | Observational | People with persistent musculoskeletal pain (n=720) | Self-management support | Rehabilitation centres, Germany |
| Mecklenburg et al (74) | RCT | People with persistent knee pain (n=162) | Self-management support (Personal coach) | USA |
| Calner et al (75) | RCT | People with persistent musculoskeletal pain (n=109) | Self-management support (Physiotherapy led) | Healthcare centres, Sweeden |
| Semrau (76) | RCT | People with persistent back pain (n=351) | Self-management support (Physiotherapy led) | Germany |
| Kelly et al (33) | Qualitative | People with persistent musculoskeletal pain (n=14), Physiotherapists (n=13) | Self-management support (Physiotherapy led) | Private, public, hospital and primary care - Ireland |
| Slater et al (99) | Observational | People with persistent back pain (n=51) | Self-management support | Remote centres, Australia |
| Johnston et al (134) | Mixed methods | People with persistent musculoskeletal pain (n=8), Vocational rehabilitation workers (n=12) | Self-management support (Online training modules) | Australia |
| Hurley et al (157) | Quasi-experimental design | Physiotherapists (n=6) | Self-management support (e-learning developed by physiotherapists) | Primary care, Ireland |
| Løchting et al (78) | RCT | People with low back pain (n=203), | Education (General practitioners and physiotherapists | Primary care, Norway |
| Bair et al (79) | RCT | People with persistent musculoskeletal pain (n=241) | Self-management support (Nurse care managers) | USA |
| Casey et al (151) | Case series or reports | People with persistent musculoskeletal pain (n=73) | Self-management support (Physiotherapist and exercise therapists) | Ireland |
| Brunnekreef et al (34) | Qualitative | People with persistent musculoskeletal pain (n=27) | Self-management support | Netherlands |
| Hutting et al (135) | Mixed methods | People with persistent musculoskeletal pain (n=31) | Self-management support (Physiotherapy led) | Netherlands |
| Van den Heuvel et al (35) | Qualitative | Physiotherapists (n=14) | Self-management support | Netherlands |
| Egerton et al (100) | Observational | People with persistent osteoarthritis of the knee (n=78) | Education | Australia |
| Barrett et al (36) | Qualitative | People with shoulder pain (n=23) | Self-management support (Physiotherapy led) | Out patient departments, Ireland |
| Johansen (37) | Qualitative | People with knee pain (n=8), Physiotherapists (n=10) | Self-management support | Australia |
| Lavender et al (38) | Qualitative | Peer mentors (n=9) | Peer support and education | UK |
| Walsh et al (80) | RCT | People with persistent osteoarthritis (n=349) | Self-management support (Physiotherapy led) | Primary care, UK |
| Shue (39) | Qualitative | Healthcare providers (n=15) (psychologist, physician, physiotherapist, nurse, social worker, pharmacist) | Peer support and education | USA |
| Najem et al (40) | Qualitative | Physiotherapists (n=8) | Education | Lebanon |
| Fioratti et al (125) | Feasibility | People with persistent musculoskeletal pain (n=65) | Self-management support | Brazil, Physiotherapy waiting lists or rehabilitation centres. |
| Gustafsson et al (41) | Qualitative | People with persistent musculoskeletal pain (n=16) | Self-management support | Sweeden |
| Perry et al (126) | Feasibility | People with persistent musculoskeletal pain (n=15) | Self-management support | Canada, waiting lists |
| Chala et al (42) | Qualitative | Healthcare providers (n=24) | Self-management support (Physiotherapist or doctor) | Ethiopia, Hospitals |
| Buchan (114) | Oral presentation abstracts | n/a | Self-management support | UK / MSK services |
| Parchment et al (136) | Mixed methods | Healthcare providers (n=71) | Education (Physiotherapist, doctor, midwife, community worker, sports professional and health improvement manager) | UK |
| Mansell et al (81) | RCT Secondary analysis | People with persistent low back pain (n=216) | Education (General practitioner and Physiotherapists) | Norway |
| Johnsen et al (101) | Observational | People with kip and knee osteoarthritis (n=22588) | Education (Physiotherapy led) | Denmark |
| Yin et al (132) | Feasibility | People with persistent musculoskeletal pain (n=38) | Self-management support (Physiotherapy led) | USA, Community centres |
| Sheppard et al (137) | Mixed methods | People with persistent musculoskeletal pain (n=122) | Self-management support (An experienced lay leader with experience of a chronic MSK condition, and a  vocational rehabilitation consultant) | Australia |
| Roberts and Busby (115) | Observational | People with persistent musculoskeletal pain (n=339) | Self-management support (Physiotherapy led) | UK community hospitals |
| Amorim et al (145) | Pilot RCT | People with persistent back pain (n=68) | Health coaching and personalised care planning | Australia, Out patient physiotherapy departments |
| Button et al (138) | Mixed methods | People with persistent musculoskeletal pain (n=48) Physiotherapists (n=15) | Self-management support (Physiotherapy led) | UK, Out patient services |
| Carr et al (102) | Observational | People with persistent musculoskeletal pain (n=98) | Self-management support (No profession listed) | UK / pain clinic |
| Meade et al (43) | Qualitative | People with persistent musculoskeletal pain (n=20) Physiotherapists (n=10) | Self-management support | UK |
| Andersen et al (77) | RCT | People with persistent musculoskeletal pain (n=141) | Self-management support (Health supervisor or Physiotherapist led) | Denmark |
| Patel et al (44) | Qualitative | Healthcare workers (n=20) (General  Practitioners n=9, physiotherapists n=10, community-based rheumatologist n=1). | Self-management support | UK, Primary care |
| Rizzo et al (45) | Qualitative | People with persistent musculoskeletal pain (n=10) | Self-management support | USA, Out patient physiotherapy clinics |
| Schütze et al (127) | Feasibility | People with persistent back pain (n=16) | Self-management support (Clinical psychology and physiotherapy led) | USA, Primary care |
| Castle (116) | Oral presentation abstracts | People with musculoskeletal conditions (n=n/a) | Self-management support (MSK self-management app) | UK, Primary care / Physiotherapy setting |
| Wang et al (158) | Quasi-experimental design | People with hip and knee osteoarthritis (n=277) | Education (Website designed by leading OA researchers,  clinicians and consumers) | Australia |
| Coutu et al (103) | Observational | People with persistent musculoskeletal pain (n=37) Occupational therapists (n=11) | Shared decision-making (Occupational therapy led) | Canada, Private and public |
| Coutu et al (139) | Mixed methods | Occupational therapists (n=11) and psychologists (n=4) | Shared decision-making | Canada, Public rehabilitation centres |
| Wellman et al (46) | Qualitative | Physiotherapists (n=6) | Education | UK |
| Van Oosterwijck et al (146) | Pilot | People with persistent musculoskeletal pain (n=6) | Education (Physiotherapy led) | Belgium |
| Galan-Martin (83) | RCT | People with persistent musculoskeletal pain (n=205) | Education (Physiotherapy led) | Spain, Primary care |
| Van Oosterwijck et al (84) | RCT | People with persistent musculoskeletal pain (n=30) | Education (Physiotherapy led) | Belgium private practice |
| King et al (47) | Qualitative | People with persistent back pain (n=11) | Education (Physiotherapy led) | UK |
| Gustavsson et al (94) | RCT Secondary Analysis | People with persistent musculoskeletal pain (n=156) | Self-management support (Physiotherapy led) | Sweden |
| Bunzli et al (48) | Qualitative | People with persistent back pain (n=9) | Self-management support (Physiotherapy led) | Ireland and Australia |
| Morris et al (49) | Qualitative | People with persistent back pain (n=6) | Self-management support (Physiotherapy led) | UK |
| Joelsson et al (50) | Qualitative | People with persistent musculoskeletal pain (n=15) | Self-management support (General practitioner led) | Sweeden, primary healthcare centres |
| Eiken et al (51) | Qualitative | People with persistent musculoskeletal pain (n=5) | Self-management support (Physiotherapy led) | Norway |
| Anderson et al (161) | Feasibility | People with hip and knee OA (n=50) | Peer support and education (Peer mentor led) | UK, Primary and secondary care |
| Horler et al (52) | Qualitative | Physiotherapists (n=5) | Education | UK |
| Hartholt et al (53) | Qualitative | Physiotherapists (n=5) | Shared decision-making | UK |
| Williams et al (117) | Oral presentation abstracts | People with musculoskeletal pain (n=11) | Designing services in partnership (Orthopaedic therapy team) | UK |
| Davies et al (104) | Observational | n/a | Education (Clinical psychologists, an occupational therapist, a  physiotherapist, and pain medicine physicians) | Australia , Pain clinics, |
| Banerjee et al (105) | Observational | People with musculoskeletal pain (n=270) | Self-management support | UK, Out patient physiotherapy |
| Patel et al (147) | Pilot cluster RCT | People with persistent back pain (n=143) | Shared decision-making (Physiotherapy led) | UK |
| Caneiro et al (152) | Case series or reports | People with persistent back pain (n=1) | Self-management support (Physiotherapy led) | Australia |
| Hurley et al (54) | Qualitative | Community organisations (n=17) | Working with communities (Trained facilitators) | UK |
| Penney and Haro (55) | Qualitative | People with musculoskeletal pain (n=41) | Self-management support (Chaplains, clinical psychologists, physiotherapists, social  workers, clerks, and medical doctor/director) | USA |
| Linden et al (85) | RCT | People with persistent back pain (n=103) | Self-management support (Physician or occupational therapist) | Germany |
| Brand et al (148) | Pilot implementation | People with osteoarthritis (n=123) | Self-management support | Australia / Osteoarthritis chronic disease management service |
| Chen et al (106) | Observational | People with persistent back pain (n=132) | Shared decision-making (SDM coaches and orthopedic  Surgeons) | Taiwan |
| Sharma et al (129) | Feasibility | People with persistent back pain (n=40) | Education (Physiotherapy led) | Nepal rehabilitation hospital |
| Lopez-Lopez et al (86) | RCT | People with persistent back pain (n=53) | Self-management support (Physiotherapy led) | Spain |
| Achten et al (56) | Qualitative | People with persistent back pain (n=12) | Self-management support (Physiotherapy led) | Netherlands |
| Adams et al (57) | Qualitative | People with persistent musculoskeletal pain (n=18) | Self-management support (No profession defined) | UK |
| Gustavsson et al (87) | RCT 2 year follow up | People with persistent neck pain (n=156) | Self-management support (Physiotherapy led) | Sweeden |
| Gustavsson et al (88) | RCT | People with persistent neck pain (n=156) | Self-management support (Physiotherapy led) | Sweeden |
| Hutting et al (58) | Qualitative | Physiotherapists and exercise therapists (n=638) | Self-management support | Netherlands |
| Hoffman et al (13) | Qualitative Survey | Physiotherapists (n=372) | Shared decision-making | Australia |
| Dando et al (118) | Oral presentation abstracts | Physiotherapists (n=54) | Shared decision-making | UK |
| NØst et al (89) | RCT | People with persistent musculoskeletal pain (n=121) | Self-management support (Physiotherapy led) | Norway |
| Burns et al (107) | Observational | People with persistent musculoskeletal pain (n=65) | Self-management support (Physician, physiotherapist and occupational therapy) | USA - Pain centre |
| Darlow et al (140) | Mixed methods | (People with OA n=19), General practitioners (n=11), primary healthcare nurses (n=14) and arthritis advocates (n= 12). | Designing services in partnership | Australia |
| Elwyn et al (90) | RCT Stepped wedge trial | People with knee osteoarthritis (n=72) | Shared decision-making (Physiotherapy led) | UK |
| Fritsch et al (160) | Intervention development | People with back pain (n=39) | Designing services in partnership (Text message designed by patients and clinicians (rheumatologists,  physiotherapists, pain specialists) | Australia |
| Osborn-Jenkins and Roberts (59) | Qualitative Secondary analysis of audio | Physiotherapists (n=25) | Education | UK |
| Farin et al (141) | Mixed methods | People with persistent musculoskeletal pain (questionnaire n=577) | Education | Germany In-patient rehabilitation |
| Clare et al (108) | Observational | People with persistent musculoskeletal pain (records n= 50) | Self-management support (clinical psychologist, physiotherapy  and clinical nurse specialist.) | UK, Primary care |
| Kohns et al (91) | RCT | People with persistent musculoskeletal pain (n=104) | Education (profession not stated) | USA |
| Chimenti et al (92) | RCT | People with achilles tendon pain (n=66) | Education (Physiotherapy led) | USA |
| McIlroy et al (60) | Qualitative service evaluation | People with persistent musculoskeletal pain (n=7), health care professionals (n=6) (Physiotherapists n=3, nurses n=2,  Psychologist n=1) | Education | UK |
| Brady et al (149) | Pilot study | People with persistent musculoskeletal pain (n=37) | Peer support and education (Mentor with lived experience of musculoskeletal  Pain) | Australia, |
| Pate et al (61) | Qualitative | People with persistent musculoskeletal pain (n=11) | Self-management support (Physiotherapist and clinical psychologist led) | Australia |
| Becker et al (93) | RCT | People with persistent musculoskeletal pain (n=44) | Health coaching and personalised care planning (Physiotherapy led) | Germany |
| Hutting et al (124) | Oral presentation abstracts | n/a | Self-management support (Physiotherapists) | Netherlands |
| Rufa et al (130) | Feasibility | People with persistent musculoskeletal pain (n=25) | Education (Physiotherapy led) | Australia |
| Parish and Ashton (120) | Oral presentation abstracts | People with persistent musculoskeletal pain (n=79) | Shared decision-making | USA |
| Stern et al (62) | Qualitative | People with musculoskeletal pain (n=31) | Self-management support (Occupational therapist and hand therapist) | USA |
| Grevnerts et al (109) | Observational | People with musculoskeletal pain (n=205), Orthopaedic surgeons (n=21) and Physiotherapists (n=60) | Shared decision-making | Sweden, Orthopaedic clinics |
| Lavender et al (63) | Qualitative | Peer Mentees (n=17) | Peer support and education | UK |
| Walston et al (153) | Case series or reports | People with back pain (n=4) | Self-management support (Physiotherapy led) | USA |
| Zimney et al (154) | Case series or reports | People with musculoskeletal pain (n=1) | Education (Physiotherapy led) | USA |
| Pattern et al (122) | Oral presentation abstracts | People with musculoskeletal pain (n=568) | Health coaching and personalised care planning | UK Physiotherapy and osteopathy waiting lists |
| Walker et al (121) | Oral presentation abstracts | People with musculoskeletal pain (n=50) | Self-management support | UK / GP practices |
| O’Hagan et al (110) | Observational | People with musculoskeletal pain (n=656) | Education | Australia / GP practices |
| Parsons et al (64) | Qualitative | People with persistent musculoskeletal pain (n=13), healthcare professionals (n=19) (Osteopaths n=5, Chiropractors n=4 and Physiotherapists n=10). | Shared decision-making | UK |
