# Supplementary material for: What does collaborative healthcare for people with musculoskeletal-related conditions look like? A scoping review
Source: BMC Musculoskelet Disord. 2025 Jul 4;26:602. doi: 10.1186/s12891-025-08814-6 (PMC12232000; doi:10.1186/s12891-025-08814-6)
Supplement: Supplementary file 2 — Supplementary Material 2 [file 12891_2025_8814_MOESM2_ESM.docx]

Supplementary file 2

Barriers to collaborative healthcare and their associated frequency count.

| **Barrier** | **Frequency count** |
| --- | --- |
| Patient values | 16 |
| Health literacy | 15 |
| Access to services | 14 |
| Psychological state | 10 |
| Levels of pain and disability | 8 |
| Inadequate training and supervision | 6 |
| Limited social support | 5 |
| Staff capability | 4 |
| Organisation of health system | 4 |
| Remote delivery | 2 |
| Level of content in professional education | 2 |
| Other responsibilities | 1 |
| Different characteristics to peer | 1 |
| Language barriers | 1 |
| Lack of support from leadership | 1 |
| Therapist centred decision making | 1 |
