# Supplementary material for: What does collaborative healthcare for people with musculoskeletal-related conditions look like? A scoping review
Source: BMC Musculoskelet Disord. 2025 Jul 4;26:602. doi: 10.1186/s12891-025-08814-6 (PMC12232000; doi:10.1186/s12891-025-08814-6)
Supplement: Supplementary file 3 — Supplementary Material 3 [file 12891_2025_8814_MOESM3_ESM.docx]

Supplementary file 3

Facilitators of collaborative healthcare and their frequency count

| **Facilitator** | **Frequency** |
| --- | --- |
| Peer support | 39 |
| Goal setting | 37 |
| Personalised approach | 34 |
| Patient values | 29 |
| Therapeutic relationships | 24 |
| Supporting dialogue | 22 |
| Problem solving | 20 |
| Diverse content | 19 |
| Communication strategies | 18 |
| Action plans | 18 |
| Self-reflection | 18 |
| Providing resources | 18 |
| Application of learning | 17 |
| Building trust | 15 |
| Promoting autonomy | 15 |
| Self monitoring | 15 |
| Social support | 15 |
| Listening | 13 |
| Patient self-tasks | 12 |
| Peer learning | 12 |
| Active learning approaches | 11 |
| Follow up plans | 10 |
| Self regulation | 10 |
| Reassurance | 7 |
| Acceptance | 7 |
| Patient readiness to change | 7 |
| Patient activation | 7 |
| Barrier identification | 7 |
| Check understanding | 7 |
| Involving employer | 6 |
| Feedback | 6 |
| Coaching techniques | 4 |
| Culturally sensitive content | 4 |
| Being responsive | 3 |
| Empathy | 2 |
| Willingness to seek support | 2 |
