# Supplementary material for: What does collaborative healthcare for people with musculoskeletal-related conditions look like? A scoping review
Source: BMC Musculoskelet Disord. 2025 Jul 4;26:602. doi: 10.1186/s12891-025-08814-6 (PMC12232000; doi:10.1186/s12891-025-08814-6)
Supplement: Supplementary file 4 — Supplementary Material 4 [file 12891_2025_8814_MOESM4_ESM.docx]

Supplementary file 4

Outcome measures capturing collaborative healthcare and their frequency count.

| Psychological  (n=77) | | Related to pain (n=68) | | Function and disability  (n=55) | | Patient knowledge and decision making  (n=33) | | Work and social (n=18) | | Performance (n=11) | | Evaluation of concept use  (n=9) | | Risk stratification (n=4) | |
| --- | --- | --- | --- | --- | --- | --- | --- | --- | --- | --- | --- | --- | --- | --- | --- |
| Pain catastrophising scale | 17 | Pain (VAS/NRS) | 28 | EQ5D | 9 | Health care utilisation | 8 | Work absence | 3 | Physical functioning (e.g., strength) | 10 | OPTION | 3 | Orebro | 3 |
| Depression or Anxiety  (Hospital Anxiety and Depression Scale)  (Patient Health Questionnaire - General Anxiety Disorder)  (The Patient Health Questionnaire Somatic, Anxiety, and Depressive Symptom Scales) | 14 | Brief Pain inventory | 9 | Roland Morris disability questionnaire | 8 | Patient activation measure | 4 | Work ability index | 3 | Step count | 1 | Health Education Impact Questionnaire | 2 | START Back | 1 |
| Tampa scale Kinesophobia | 11 | Pain self-efficacy | 9 | Oswestry disability index | 5 | Health literacy (Health Literacy Measurement Scale)  (REALM-R instrument) (Health Literacy Questionnaire) | 4 | Work limitations questionnaire | 2 |  |  | Communication evaluation in rehabilitation tool | 1 |  |  |
| Fear avoidance beliefs questionnaire | 11 | Pain Coping (Coping strategies questionnaire)  (Pain coping Inventory) | 9 | SF-36 | 5 | Client satisfaction questionnaire | 4 | Education (Self-reported) | 2 |  |  | Comprehensibility of health education programs | 1 |  |  |
| General self-efficacy scale | 6 | Pain related disability | 7 | Patient specific functional scale | 4 | Patient global impression of change | 3 | Participation and autonomy at work | 2 |  |  | Patient education materials and assessment tool | 1 |  |  |
| Arthritis self-efficacy | 3 | Pain vigilance and awareness questionnaire | 2 | Neck Disability Index | 4 | Pain stages of change questionnaire | 3 | Multidimensional scale of perceived social support | 2 |  |  | Shared decision-making process questionnaire | 1 |  |  |
| Burnout (Maslach Burnout Inventory) | 3 | Neurophysiology of pain questionnaire | 2 | Illness perception questionnaire | 3 | Partners in health scale | 2 | Stanford Presenteeism scale | 2 |  |  |  |  |  |  |
| Negative mood regulation scale | 2 | Graded Chronic Pain Scale | 1 | PROMIS | 3 | Patient goal priority questionnaire | 1 | Employment status (self-reported) | 2 |  |  |  |  |  |  |
| Self-reflection and insight scale | 2 | Multidimensional Pain Inventory | 1 | Disability of Arm Shoulder and Hand | 2 | Control preference scale | 1 |  |  |  |  |  |  |  |  |
| Chronic pain acceptance questionnaire | 2 | Centrality of pain scale | 1 | Short musculoskeletal function assessment | 2 | Decision self-efficacy | 1 |  |  |  |  |  |  |  |  |
| Back beliefs questionnaire | 2 |  |  | SF-12 | 2 | Decision quality Instrument | 1 |  |  |  |  |  |  |  |  |
| Self-efficacy and exercise health beliefs questionnaire | 1 |  |  | Patient generated index | 1 | Health Evaluation Impact Questionnaire | 1 |  |  |  |  |  |  |  |  |
| The 13-item Norwegian version of the Sense Of Coherence scale | 1 |  |  | Injustice experience questionnaire | 1 |  |  |  |  |  |  |  |  |  |  |
| Stress (Perceived Stress Scale) | 1 |  |  | Focus on therapeutic outcome low back functional status PROM | 1 |  |  |  |  |  |  |  |  |  |  |
| Post traumatic stress Post-traumatic Stress Disorder Checklist | 1 |  |  | Knee Injury and Osteoarthritis Outcome Score | 1 |  |  |  |  |  |  |  |  |  |  |
|  |  |  |  | Western Ontario and McMaster Universities Arthritis Index | 1 |  |  |  |  |  |  |  |  |  |  |
|  |  |  |  | Health related quality of life | 1 |  |  |  |  |  |  |  |  |  |  |

Key: VAS=visual analogue scale; NRS=numerical rating scale; REALM-R=rapid estimate of adult literacy in medicine revised; PROMIS=patient reported outcome measurement information system; SF=short form; PROM=patient reported outcome measure;
